# Molecular Dynamics-Instantaneous Frequencies of Molecules Method to Address the Performance of Classical and Rigid Water Force Fields

**DOI:** 10.1021/acs.jpcb.5c08265

**Published:** 2026-04-29

**Authors:** Nicolas Molina Trujillo, Laura X. Sepulveda-Montaño, Daniel G. Kuroda, Johan F. Galindo

**Affiliations:** † Department of Chemistry, 28021 Universidad Nacional de Colombia sede Bogotá, 111321 Bogotá, Colombia; ‡ Department of Chemistry, 5779 Louisiana State University, Baton Rouge, Louisiana 70803, United States

## Abstract

The accurate parametrization of molecular force fields (FFs) is crucial for obtaining reliable descriptors from molecular dynamics simulations. Benchmarking FFs requires methods that test directly or indirectly the changes in interaction potential caused by fluctuations in the chemical environment. This work uses the *Molecular Dynamics-Instantaneous Frequencies of Molecules* (MD-IFM) method as a tool for FF benchmarking, which allow us to compute vibrational spectroscopic observables, including central frequencies (ω_0_), frequency fluctuation amplitudes (σ), and spectral diffusion correlation times (τ_c_), from MD simulations. We demonstrate the utility of this method by evaluating the vibrational descriptors obtained for common water FFs (TIP3P, SPC/E, TIP4P/Ew, TIP5P, and OPC) against the high-level MB-pol potential, as well as how these descriptors are useful for addressing solvation structure and dynamics. While the classical FFs performed well in predicting vibrational frequencies of water, they systematically underestimated the heterogeneity of the local environment (σ) and show faster spectral diffusion dynamics (lower τ_c_ values) compared to MB-pol. Analysis suggests that these shortcomings are linked to a simplistic representation of the hydrogen-bonding network. In addition, our results validate MD-IFM as an efficient and robust approach for FF validation.

## Graphical abstract

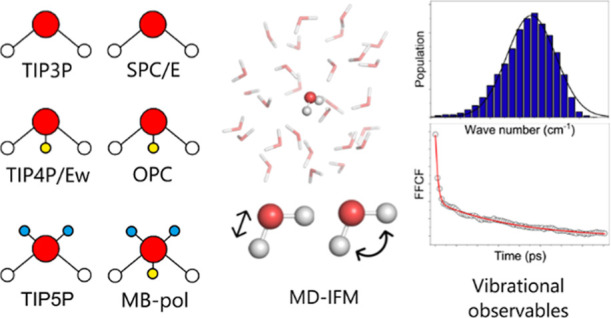

## Introduction

The accurate description of the structure and dynamical behavior of molecular systems is fundamental to understand their bulk properties, such as density, boiling and melting points, conductivity, and many others.^1,2^ Therefore, the characterization of the molecular structure, solvation dynamics, and diffusion dynamics is of great importance. For instance, the slow diffusion dynamics and strong intermolecular interactions in battery electrolytes generally lead to highly viscous systems with low conductivity and poor transport properties.^3^ Another example is enzymes, which require a precise arrangement of the amino acids in the active site to bind the substrate and to stabilize the transition state, since slight changes in the substrate orientation considerably alter the reaction kinetics of the catalytic process.^4,5^ Furthermore, the multicomponent nature of complex solutions (i.e., deep eutectic solvents and ionic liquids among others) can result in the presence of nanoheterogeneities that can affect the ability to solvate solutes.^6^

Experimentally, spectroscopic techniques are widely used to investigate the molecular structure (i.e., scattering techniques) and dynamics (e.g., time-resolved techniques) of complex systems.^7–22^ Nonetheless, the signals and metrics derived from these experimental techniques are generally difficult to interpret. Thus, computational modeling is commonly used in conjunction with experiments to shed light on the molecular scale behavior of the system.^23–57^ An accurate description of the potential energy surface (PES) is therefore required to produce predictive molecular dynamics simulations. To this end, the PES can be built from ab initio methods, semiempirical methods, or classical force fields (FFs).

First-principles and semiempirical methods provide a more accurate description of the system, but they are computationally more expensive than classical FFs. For example, even with the use of supercomputers, these calculations are feasible only for a few hundreds of atoms with time scales limited to picoseconds in the best-case scenario.^58^ Contrastingly, the classical representations of molecular systems given by classical FFs^59^ are faster and computationally more efficient, making them widely used in modern large-scale molecular simulations. However, the ability of these methods to correctly represent the intricacy of PES is in many cases limited. Hence, a methodology capable of benchmarking FFs is required to assess the suitability of the molecular representation of the system, thereby ensuring that the FF can be confidently used to generate molecular dynamics simulations, from which information can be extracted to interpret the experimental results.

Different approaches exist to benchmark computational methods; some examples include calculation of the energetics of phase transitions or solvation and its subsequent comparison with experiments or higher levels of theory.^60–64^ Typically, the benchmark tests the shape (i.e., minimum and concavity) of the PES produced by the FF.^65–67^ However, these characteristics can also be derived from the fluctuations in the PES^68^ observed in molecules due to intermolecular interactions.^69^ Both the shape and fluctuations of the PES are classically explored by using FFs through molecular dynamics (MD) simulations. Therefore, a benchmarking method can indirectly test the shape of the PES through dynamic fluctuations of its chemical environment. To this end, observables capable of characterizing the shape and fluctuations of the PES are essential.

Vibrational observables, such as central frequencies (ω_0_), frequency fluctuation amplitudes (σ), and decorrelation times (τ_c_) from vibrational spectral diffusion, are valuable metrics that describe the properties of the PES. While the first two metrics provide information about the ensemble average structure of a molecule and the molecular environment experienced by it, on average, the latter describes the dynamics of the system through the loss of vibrational frequency correlation.^54,70^ Experimentally, these quantities are readily obtained using a combination of linear and nonlinear spectroscopic techniques such as Fourier-transform infrared spectroscopy (FTIR) and two-dimensional infrared spectroscopy (2DIR) or the three-pulse photon echo method.^69^ Hence, vibrational spectroscopy and its observables make it possible to perform a one-to-one comparison between experiments and molecular simulations.

Frequency maps^71^ have been widely used to simulate vibrational observables computationally. However, frequency maps usually require extensive parametrization, and their transferability to different solvents and more heterogeneous environments is not always assured.^72–74^ Other approaches^38,54,75–77^ exist for translating molecular coordinates to vibrational observables from which one can calculate the spectral line-shape, the peak shift, or frequency–fluctuation correlation functions (FFCF).^78^ However, these approaches often increase the computational cost. Herein, the *Molecular Dynamics-Instantaneous Frequencies of Molecules* (MD-IFM)^58,79^ is used as a benchmarking tool for FFs. The MD-IFM is a parametrization-free method developed to calculate the aforementioned vibrational spectroscopy observables from a MD simulation. In addition to avoiding complex parametrization, the MD-IFM computational cost is low, comprising classical molecular dynamics and frequency calculations with a semiempirical level of theory using GFN2-xTB.^80^ Comparatively, DFT computations are more than a thousand times slower than GFN2-xTB to achieve the same calculations.^79,80^ Furthermore, the MD-IFM has been shown to accurately reproduce the amide I mode of *N*-methylacetamide and the C–H stretching of chloroform in simple and complex solvents using this computational framework.^58,81^ Given that the accuracy of MD-IFM relies on an adequate description of the PES, it serves as a unique tool to assess the performance of water FFs.

Molecular simulations involving water are common, and many FFs have been developed for this purpose, including common classical water models, such as 3-site models (TIP3P^82^ and SPC/E^83^), which assign fixed charges to the oxygen and hydrogen atoms. These classical FFs offer a good balance between computational efficiency and accuracy^84^ but do not account for changes in charge distributions in response to an external electric field (polarization). Also, more complex FFs including TIP4P/Ew,^85,86^ OPC,^87^ and TIP5P^88^ introduce additional charges at “dummy” sites to better capture electrostatic properties and multipole effects and modulate the exchange interaction to improve the accuracy of exchange and dispersion energy calculations.^89^

Other FFs such as AMOEBA^90,91^ use induced point dipoles and multipole representations to introduce polarization effects. For example, the CHARMM Drude model^92^ adds Drude particles to the atoms for the same purpose. TIP4P/PQ^93^ and POL3 ^94^ are also water-specific polarizable models, which are widely used in systems with high electronic environment like ionic solvation or water–metal interfaces.^95,96^ While polarizable models account for the dynamic response of the charge distribution to external electric fields and give more chemically accurate simulations than fixed-charge models, they are not widely adopted as the nonpolarizable FFs due to their high computational cost.

Lately, MB-pol has been developed to accurately represent water. MB-pol is a particularly important force field^97–99^ and perhaps the closest to being considered a universal model due to its ability to reproduce water properties in both gas and condensed phases,^100^ without relying on separate models (e.g., TIP4P/Ice^101^ used for ice simulations). The many-body potential nature of MB-pol makes it capable of accurately describing many structural and thermodynamic properties, some of them with subchemical accuracy.^102^ Despite neural network potentials being a promising point of reference 103,104 and ab initio methods yielding overall better results, we chose MB-pol as a gold standard in the evaluation of other water models through vibrational frequencies due to the extensive validation of its ability to reproduce vibrational spectra of water clusters, infrared and Raman spectra of liquid water, the infrared bands for H_2_O and HDO water probes, and the spectral diffusion process of the OD stretching mode in the 2DIR spectra.^47,50,52,105–107^ However, MB-pol, like polarizable FFs, is limited to relatively short molecular dynamics simulations, making the description of the large molecular systems, like proteins, in water for hundres of nanoseconds prohibitively expensive.^108,109^

In this work, our aim is to showcase the MD-IFM method as a tool to address the aforementioned vibrational observables of an HDO molecule in a bath of explicit water molecules represented with different FFs (TIP3P,^82^ TIP4P/Ew,^85,86^ TIP5P,^88^ SPC/E,^83^ and OPC^87^), using MB-pol as a gold standard in describing water solvation environment and dynamics.

## Methods

The MD-IFM method consists of prior exploration of the configuration space through classical molecular dynamics to later postprocess a large group of solvent configurations by computing their vibrational frequencies one-by-one using a semiempirical method that has shown to calculate accurately enough vibrational frequencies. To conserve the PES exploration, a geometry optimization of the molecular probe within the chemical environment described by the FF is performed. All of the choices such as cluster size, sample length, etc., were carefully chosen after metric convergence, allowing us to attribute the obtained results to FFs performance rather than other sources.

### Molecular Dynamics (MD) Simulations

MD simulations for each selected FF were carried out using the AMBER24 package.^110^ Water boxes with a side length of 40 Å were built using the PACKMOL software.^111^ An energy minimization was performed prior to heating over 100 ps from 0 to 300 K in the *NVE* ensemble; then, an equilibration step over 1 ns in the *NPT* ensemble was performed followed by the production step for which 12 replicas of 60 ps in the *NVE* ensemble were calculated. The last 50 ps of the production run were used for the subsequent frequency calculations, extracting snapshots every 50 fs for a total of 12,000. The integration time step for Newton’s equations was set to 2 fs in all stages. SHAKE was employed to constrain bonds involving hydrogen atoms. For MB-pol, snapshots were taken every 48 fs.

### Frequency Calculations

For the frequency calculations, a cluster was extracted for each snapshot by selecting all water molecules within the first three solvation shells around a fixed water molecule acting as a molecular probe, which is based on the radial distribution function (Figure S1), which resulted in a cluster of 30 molecules. We also calculated the number of water molecules in the first solvation shell of the chosen probe for each system; for this purpose, the first minimum of the radial distribution functions was taken as the distance cutoffs. Optimization of only the central molecular probe, for which one hydrogen was substituted by deuterium, and frequency calculations were carried out with the GFN2-xTB algorithm as previously reported,^58^ conserving the sampled geometries of the remaining water molecules of the cluster as described by the FF and the influence of this environment on the vibrational frequencies of the HDO molecule.

The values of ω_0_ corresponding to each vibrational mode of the HDO probe were obtained as the intensity-weighted average of the frequencies. Frequency distributions were also constructed using the resulting 12,000 frequency values, and σ was obtained from the intensity-weighted standard deviation of the frequencies. The FFCF was calculated as the autocorrelation of the frequencies over time (*C*(*t*) = <δω­(*t*)­δω­(0)>), where δω­(*t*) represents the difference between the frequencies at time *t* and the average frequency along the trajectory, following the Kubo formalism.^69^ The FFCF was modeled with biexponential decays ( $C\left(t\right)=A_{1}e^{-t/\tau_{1}}+A_{2}e^{-t/\tau_{c}}+y_{0}$ )^54^ to obtain the τ_*c*_ for each normal mode as the longest characteristic time.

Standard errors for the uncertainty calculations were taken directly from fitting parameters or computed as $SE=\frac{s}{\sqrt{N}}$ , *s* being the standard deviation or weighted standard deviation (for the central frequencies). Reported values of uncertainty correspond to 1.96 times the standard error based on a 95% confidence interval.

## Results and Discussion

The molecular environment observed on average by a water (HDO) molecule and its dynamics were evaluated from MD simulations with the different FFs using the values of central frequencies (ω_0_), frequency fluctuation amplitudes (σ), and decorrelation times (τ_*c*_) for vibrational spectral diffusion. These values were computed for the three vibrational modes of the HDO molecule: HOD bending, OD stretching, and OH stretching. The results from the MD-IFM method for the vibrational observables for each vibrational mode are presented in Table 1, and each of the individual metrics is discussed separately in the following sections.

**Table 1:** General Results of ω_0_, σ, and τ_*c*_ Obtained through the MD-IFM Method for the Evaluated FFs and HDO Vibrational Modes

force field	HOD bending			OD stretching			OH stretching		
	ω_0_ (cm^–1^)	σ (cm^–1^)	τ_*c*_ (ps)	ω_0_ (cm^–1^)	σ (cm^–1^)	τ_*c*_ (ps)	ω_0_ (cm^–1^)	σ (cm^–1^)	τ_*c*_ (ps)
TIP3P	1350.2 ± 0.5	26.0 ± 0.3	0.23 ± 0.02	2520 ± 2	70 ± 5	0.39 ± 0.01	3468 ± 2	95 ± 7	0.43 ± 0.03
SPC/E	1339.8 ± 0.5	27.4 ± 0.5	0.86 ± 0.07	2473 ± 2	82 ± 6	0.80 ± 0.04	3406 ± 2	115 ± 7	0.65 ± 0.03
TIP4P/Ew	1348.5 ± 0.5	25.5 ± 0.6	0.40 ± 0.03	2498 ± 2	75 ± 5	0.80 ± 0.05	3418 ± 2	109 ± 6	0.83 ± 0.05
OPC	1341.2 ± 0.5	24 ± 1	0.44 ± 0.07	2551 ± 1	63 ± 2	0.64 ± 0.02	3507 ± 2	85 ± 4	0.85 ± 0.04
TIP5P	1346.1 ± 0.5	25.8 ± 0.6	0.95 ± 0.05	2488 ± 2	82 ± 8	0.69 ± 0.02	3426 ± 2	110 ± 10	1.09 ± 0.03
MB-pol	1340.9 ± 0.6	28.9 ± 0.7	0.51 ± 0.04	2470 ± 2	82 ± 5	1.21 ± 0.06	3403 ± 2	115 ± 8	1.34 ± 0.08

### Central Frequencies (ω_0_)

The ω_0_ accounts for the influence of the chemical environment and its average structure, allowing us to evaluate the overall PES description made by FF. The values of ω_0_ for the HOD bending mode range from ∼1339 to ∼1350 cm^–1^ (Table 1), showcasing the similar performance of all FFs. However, these values are underestimated when compared to a reported experimental value of 1458 cm^–1^.^32^ Furthermore, SPC/E and OPC yield a value very close to that of MB-pol (Figure 1A), while each of the other FFs differs by a small percentage, ranging between 0.4% and 0.7%. For the OD stretching mode, the ω_0_ values are more disperse in the range from ∼2470 cm^–1^ to ∼2551 cm^–1^ (Table 1). While the OD stretches have a broader dispersion in the predicted central frequencies than that observed for the HOD bending mode, the values remain in a relatively small range (i.e., errors <3.3%). However, in this case, all the classical FFs show major deviations from MB-pol, except for SPC/E. The latter has a central frequency with an error of only 0.1%, while others showed deviations up to 3.3% (Figure 1A), but relative to the experimental value of ∼2500 cm^–1^,^112^ TIP4P/Ew produces the closest result. The OH stretching mode shows the expected frequency shift from the OD stretching, and its central frequencies appear in the range between ∼3403 cm^–1^ and ∼3507 cm^–1^, but with similar deviations to that of the OD stretching mode. The percentage differences relative to MB-pol again vary between 0.1% and 3.0%, with SPC/E showing the lowest and OPC the largest deviations (Figure 1A), and this time, MB-pol result is the closest to the experimental value of ∼3400 cm^–1^.^55,112^ It is worth noting that the outstanding performance of SPC/E could be related to its effective polarization correction,^83^ included to account for a more realistic dipole moment of water molecules that may result in a better representation of water average structure and consequently a more accurate central frequency.

**Figure 1:**
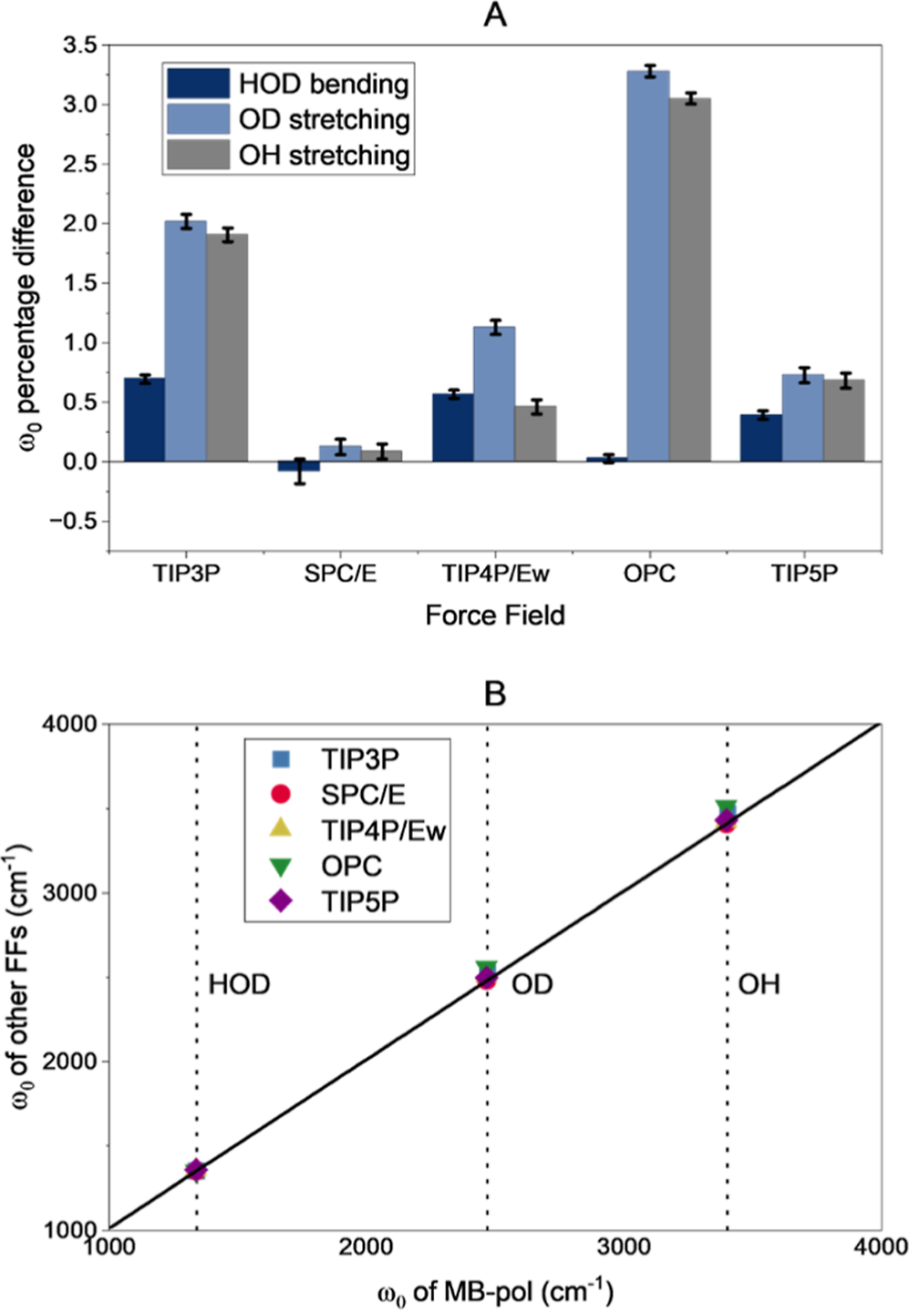
(A) Percentage differences of ω_0_ predicted with the classical FFs with respect to MB-pol. Differences greater than zero indicate that MB-pol yielded a lower value and vice versa. Error bars crossing the *x* axis denote negligible differences between the values. (B) Correlation plot of ω_0_ predicted with the classical FFs versus the one predicted with MB-pol. The black line represents a *y* = *x* function and vertical dotted lines are the position of the vibrational modes for the MB-pol FF.

A better representation of the performance of the different classical FFs is obtained by directly comparing their predicted central frequencies for the different HDO modes for each classical FF against MB-pol (Figure 1B). In this representation, larger errors are seen as larger distances from the diagonal line. The results show that all FFs have small deviations, with OPC having a small, but more noticeable distance than the rest. Moreover, the predictions of the OD and OH stretching frequencies are less precise than those of the bending mode. Overall, all the FFs produce HDO vibrational central frequencies with reasonable accuracy compared to those of MB-pol.

As previously mentioned, the central frequencies of the OD and OH stretching modes are less precise than those predicted for the bending mode of the same molecule. This difference can be attributed to the different sensitivity of each water vibrational mode to changes in the chemical environment, or equivalent, the hydrogen-bonding state of the water molecules.^54,55^ For example, water molecules with a low number of hydrogen bonds or in the gas phase exhibit a blueshift in the vibrational frequencies of their vibrational modes as compared to water molecules in a tetrahedral hydrogen-bond network.^113^ Hence, a precise description of the molecular interactions, hydrogen bonds in this case, by the FF should result in a small deviation of the computed central frequency. This also provides a reliable metric for evaluating the quality of the FF when compared to that of the gold standard. To assess the effect of the solvation shell into the vibrational mode frequencies for each FF, the central frequency of each HDO mode was computed as a function of the number of water molecules in the first solvation shell (the chosen values were 3, 4, and 5 for all the FFs and also 6 for SPC/E, since its population was comparable to that of 3, Figure S2).

The central frequency results, presented in Figure 2, show that the classical FFs reproduce the expected shift for the vibrational frequencies as a function of the number of water molecules in the first solvation shell (solvatochromism), where a higher number of water molecules indicates a higher probability of hydrogen-bond formation. In the case of the HDO bending mode, the TIP4P/Ew, OPC, TIP5P, and MB-pol FFs showed a continuous decrease in the central frequency as a function of the number of water molecules, while SPC/E and TIP3P have an overall decrease but are not monotonous. Moreover, the range of central frequencies is small (∼3 cm^–1^) in agreement with the bending mode being less sensitive to the chemical environment than the water stretching modes.^32^ For both stretching modes of HDO, the central frequencies show the expected large range in variations of ω_0_ (∼25 cm^–1^) as a function of the number of water molecules consistent with the higher sensitivity of these modes to the hydrogen-bond environment. Remarkably, for the HDO stretches, all FFs, but TIP5P, consistently reproduced the expected redshift of the central frequency with increasing number of water molecules (Figure 2B,C), indicating that most FFs correctly represent the impact of the hydrogen-bonding state of water molecules on the central frequency.

**Figure 2:**
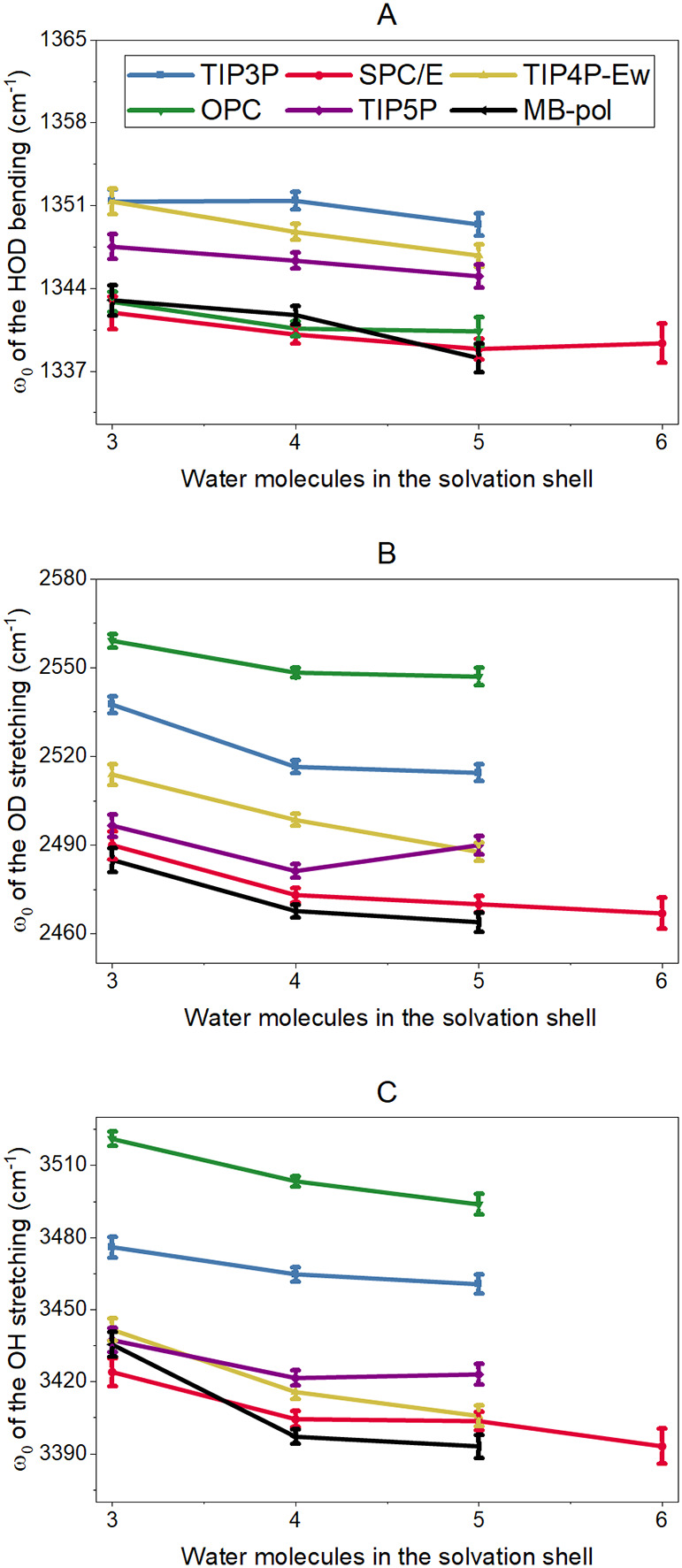
ω_0_ values as a function of the number of water molecules in the first solvation shell of the probe for the (A) HOD bending, (B) OD stretching, and (C) OH stretching modes.

### Frequency Fluctuation Amplitudes (σ)

Frequency distributions and their shapes are useful for describing the heterogeneity of the conformational space observed by the HDO molecule in the MD simulation. Since σ represents the standard deviation of the frequency distributions, a higher σ value indicates a larger variance in the frequency values, which are tightly related to the variability of the probe chemical environment. These values could be compared to some extent to the line width of IR bands or be derived from the pre-exponential factors of the FFCF according to the Kubo formalism.^69^

The results show that the HOD bending modes have distributions close to a single Gaussian (Figure S3 and Table S1), indicating the stochastic behavior of frequency fluctuations for this water mode, while the stretching modes (Figures S4 and S5 and Tables S2 and S3) have distributions which are not represented with a single Gaussian, indicating a more complex vibrational dynamic.^114^ Specifically, the amplitude of frequency fluctuations (σ) for the HOD bending mode ranges from ∼24 to ∼29 cm^–1^ (Table 1) in all FFs. These values of σ are significantly different from those of MB-pol (Figure 3A). Notably, MB-pol presents the broadest frequency distribution for the HDO bend, while OPC shows the narrowest with the rest falling in between. The closest description of the HOD bending with respect to MB-pol is given by SPC/E, which has a difference of only −5.1%. All the other FFs showed large deviations, up to −16.4%. In the case of the OD stretching mode, the σ values are much larger, ranging from 63 cm^–1^ to 82 cm^–1^. However, the percentage differences relative to MB-pol are similar to those described in the bending mode (i.e., −0.3% to −23.8%). Moreover, both SPC/E and TIP5P do not present significant differences from MB-pol, while OPC has the largest deviation due to its narrower frequency distribution. Finally, the OH stretching mode exhibits the widest distributions, with σ = 85 – 115 cm^–1^, with the percentage differences with respect to MB-pol ranging from −0.3% to −26.5% and following a similar pattern to that of the OD stretching mode; i.e., where SPC/E, TIP4P/Ew, and TIP5P are the closest to MB-pol and the OPC with its narrow distribution has the largest difference.

**Figure 3:**
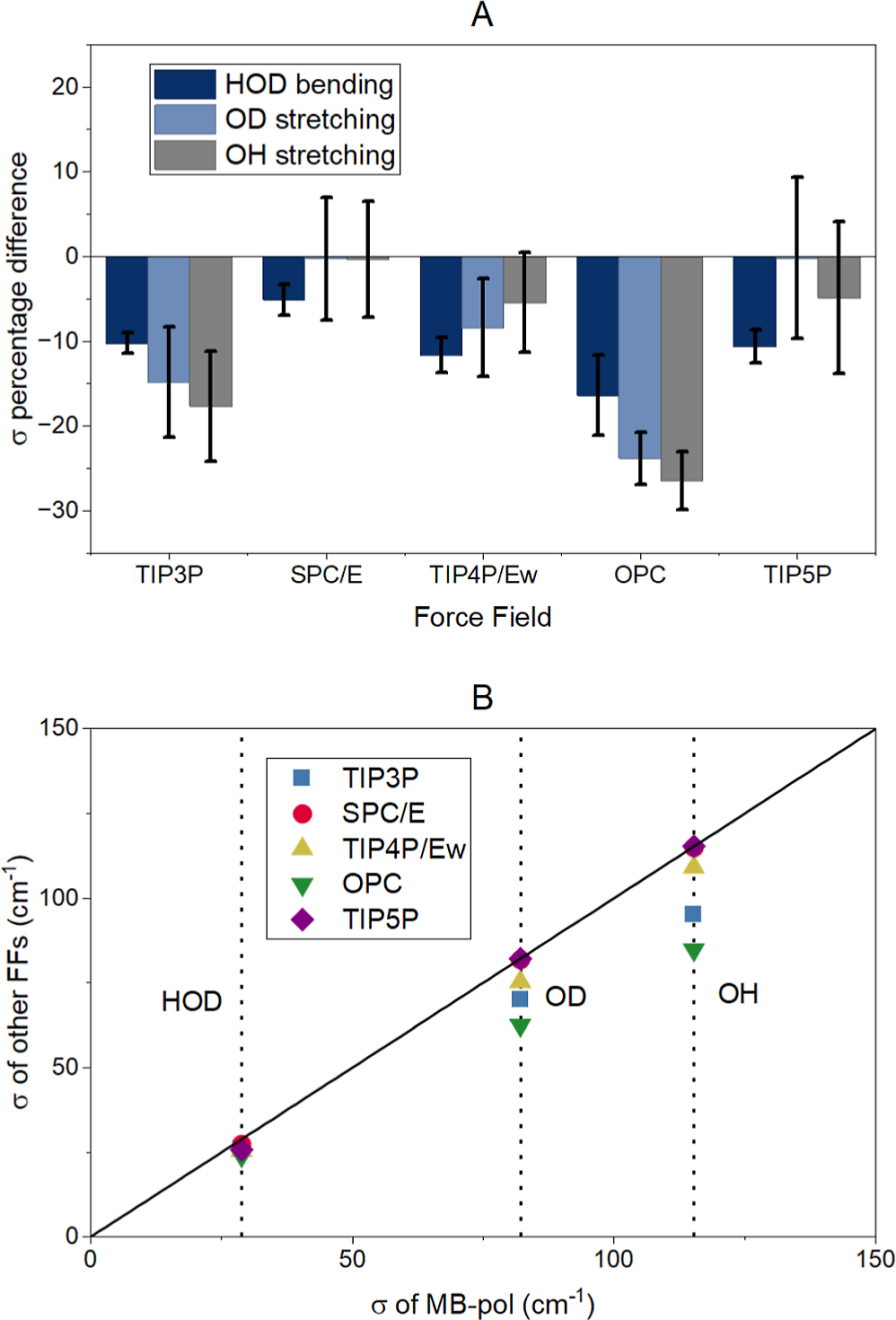
(A) Percentage differences of σ predicted with the classical FFs with respect to MB-pol. Differences greater than zero indicate that MB-pol yielded a lower value and vice versa. Error bars crossing the *x* axis denote negligible differences between the values. (B) Correlation plot of σ predicted with the classical FFs versus the one predicted with MB-pol. The black line represents a *y* = *x* function and vertically dotted lines the vibrational modes.

The correlation plot in Figure 3B summarizes the results of the distribution widths for all classical FFs when compared to MB-pol. It is evident from these results that all FFs predict narrower frequency distributions than MB-pol. While this underestimation appears to be more pronounced for the two HDO stretching modes, the errors with respect to MB-pol show statistically similar numbers for all modes (Figure 3A), except for TIP3P and OPC with statistically different results.

A consistent result in the frequency distributions of all FFs is the tendency of OPC and TIP3P to produce narrower distributions for the HDO stretching modes. Analysis of the number of water molecules in the first solvation shell of the HDO molecule (Figure S2) shows that the OPC FF produces an environment with a slightly less heterogeneous hydrogen-bonding environment for the water molecules. However, this is not the case of TIP3P since it has a broader distribution of hydrogen-bond states (Figure S2). Therefore, vibrational frequency distributions computed from the OPC-MD have smaller fluctuations because the HDO molecule experiences a less diverse number of chemical environments in the simulation (Figure S2). In contrast, the case of TIP3P cannot be simply explained with the same hydrogen-bonding arguments (Figure S2), but it is likely related to a less heterogeneous environment observed by the HDO molecules in the simulation.

While models, like TIP3P and TIP4P/Ew, use an O–H bond length close to the experimental gas-phase value, the OPC utilizes a significantly shorter bond length of 0.8724 Å and a narrower H–O–H angle of 103.6°. This water molecular structure is designed to optimize the molecular quadrupole moment for bulk dielectric properties^87^ but may alter the local electrostatic landscape around the probe. Hence, the OPC is an FF optimized for macroscopic bulk properties, which sacrifices its accuracy in describing the microscopic heterogeneity of the local solvation environment.

The effect of the solvation structure on the shape of the frequency distributions was also examined by evaluating the change in the distributions as a function of the number of water molecules in the first solvation shell of HDO (the same values used for ω_0_ analysis). In the case of the HOD bending, the underlying distributions not only are well represented by a single Gaussian but also have their maxima at approximately the same frequency (Figure 4A and Table S4). This latter observation is consistent with the central frequency results and the lack of solvatochromism of the water bending mode as previously reported.^32^ Moreover, their asymmetry coefficients (ACs), as representative of their skewness, are relatively small, in agreement with their Gaussian shape. In contrast, the HDO stretching modes have underlying distributions with noticeable redshifts (Figure 4, Tables S6 and S8) directly related to the high sensitivity of the stretch mode to the number of water molecules in the first solvation shell. Additionally, all the subdistributions are asymmetrical and exhibit negative skewness, as revealed by their ACs (Tables S5, S7, and S9). Interestingly, the ACs are considerably larger for the stretching modes of HDO than their bending analogue. In addition, in all cases, the ACs are comparable with MB-pol except for OPC, which is smaller. Again, the large asymmetries in the frequency subdistributions reflect the impact of hydrogen-bond state to the HDO molecule. This effect is readily seen in the distributions of both OD and OH stretching modes when the HDO molecule has three water molecules in the first solvation shell (Figure 4). In this case, all frequency distributions present consistently higher skewnesses than those corresponding to more solvated water molecules. Molecularly, the large asymmetry is likely caused by the sparse spatial distribution of a small number of water molecules, since small geometrical changes in this solvation shell should lead to substantial frequency fluctuations. The asymmetry of the frequency distributions observed here agrees with previous studies,^32,113^ where they were assigned to the populations of water in distinct hydrogen-bond states. It is important to note that the redshift and asymmetry in the distributions are consistently reproduced by all the FFs and MB-pol giving a strong validation to their existence.

**Figure 4:**
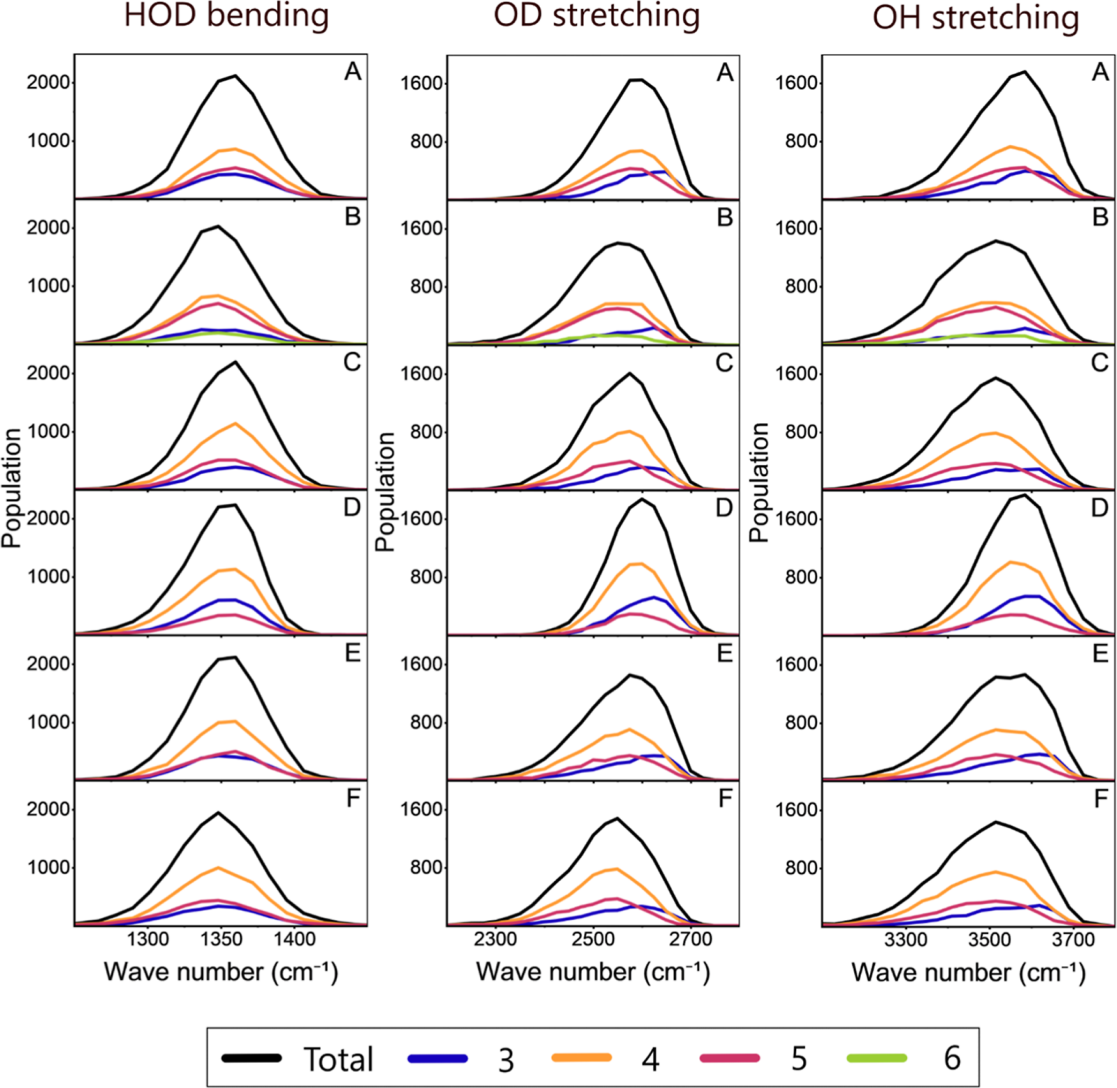
Frequency distributions of the HOD bending mode, OD stretching mode, and OH stretching mode obtained for the simulations using TIP3P (A), SPC/E (B), TIP4P/Ew (C), OPC (D), TIP5P (E), and MB-pol (F). There is also represented the contribution of subdistributions related to different numbers of water molecules in the first solvation shell of the probe.

### Spectral Diffusion (τ_*c*_)

In contrast to the previous observables (ω_0_ and σ), vibrational spectral diffusion and its associated characteristic times are dependent on the dynamics of the molecular environment. Hence, this metric provides an estimate of the time scale of structural changes occurring in the MD simulation and is experimentally measured from the 2DIR spectra with methods, such as CLS.^78,115^ In other words, the dynamics of spectral diffusion depict the time evolution of underlying molecular processes occurring in the liquid responsible for the loss of the memory of a state over time, or equivalent, the dynamics of the decorrelation of vibrational frequencies.^69^ In here, spectral diffusion represents the autocorrelation of the fluctuations of the water vibrational frequencies due to the time evolution of the solvation shell.

For water, it has been reported that the loss of vibrational frequency correlation is associated with a fast hydrogen-bond breaking and making in the femtosecond time scale (50–400 fs) and a slower hydrogen-bonding network reorganization in the picosecond time scale (0.7–1.2 ps).^54,55,116^ This is a molecular phenomenon successfully reproduced by MB-pol.^50^ Here, the dynamics of the spectral diffusion was computed from the FFCF, where each FFCF is described with biexponential decay functions (see Figures S6–S8 and Tables S10–S12) to obtain the characteristic times.

All classical FFs (Table 1) qualitatively reproduce the expected biexponential decay behavior of water (*R* ^2^ > 0.97, see Figures S6–S8 and Tables S10–S12) in agreement with the fast and the slow components of the water spectral diffusion.^13–22^ However, the analysis here is only focused on the picosecond component, as it can be directly compared to experimental values obtained from 2DIR spectroscopic experiments. Figure 5A shows the percent differences and relative to MB-pol of the τ_*c*_ values for the hydrogen-bond network reorganization as a function of both the classical FFs and the HDO vibrational mode. For the HOD bending mode, the OPC yielded the closest τ_*c*_ to MB-pol with an error of −13.0%, while the other FFs showed larger differences, ranging from −54.9% to 86.3%. Similarly, both stretching modes have picosecond decorrelation times with deviations ranging from −18.7% to −67.9% with respect to MB-pol. However, in the stretching cases, the characteristic time of the hydrogen-bond network rearrangement for all FFs was observed to be smaller when compared to MB-pol. Moreover, TIP4P and TIP5P consistently yield closer values than the rest FFs for the stretching modes. It is important to note that both hydrogen atoms of the HDO molecule were treated equally during the MD simulations, and so, it was expected to observe the same dynamical behavior for both OD and OH stretching modes. However, this is not the case for all the FFs, only TIP3P, TIP4P/Ew, and MB-pol have comparable values of τ_*c*_, indicating that the conformational space exploration might have not been sufficient. This result is surprising given that 250 ps dynamics were used for their conformational space sampling, which should have been sufficient to account for the description of the picosecond time scale of water spectral diffusion.

**Figure 5:**
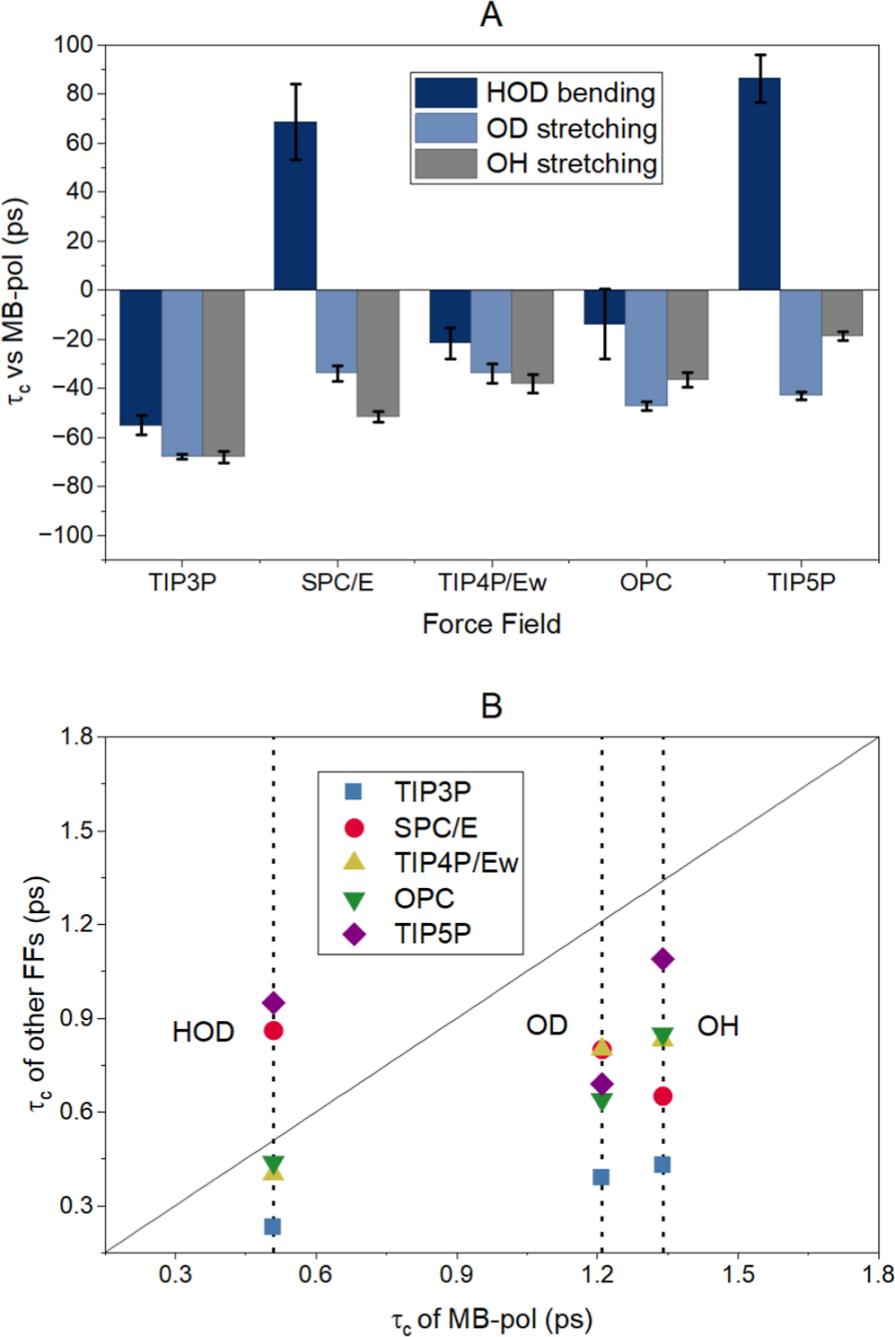
(A) Percentage differences of τ_*c*_ predicted with the classical FFs with respect to MB-pol. Differences greater than zero indicate that MB-pol yielded a lower value and vice versa. Error bars crossing the *x* axis denote negligible differences between the values. (B) Correlation plot of τ_*c*_ predicted with the classical FFs versus the one predicted with MB-pol. The black line represents a *y* = *x* function and vertical dotted lines the vibrational modes.

Further comparison of the spectral diffusion dynamics is obtained from the correlation plot (Figure 5B) between MB-pol and classical FFs. The plot shows significant scattering in the computed values, where most FFs yield τ_*c*_ values smaller than those of MB-pol. This result indicates that classical FFs are prone to produce faster dynamics in the chemical environment around the HDO molecule. This phenomenon could be related to the models using a completely rigid water molecule, which account for rotational and translational degrees of freedom, but neglects more refined microstructure motions. More complex FF like the 4-site and 5-site models explicitly separate the negative charge from the oxygen center, allowing for a more realistic representation of the tetrahedral hydrogen-bond network when compared to 3-site models. Hence, the charge concentration on the oxygen atom used in the 3-site models can artificially flatten the potential energy surface, a feature that may impact the description of local structure and dynamics, leading to a worse performance compared to MB-pol.

Overall, all the nonpolarizable classical FFs do not produce quantitative predictions of the spectral diffusion as seen by the underestimation of the decorrelation times and errors larger than 20% when compared to the value produced by MB-pol. However, even if the time scales are not very accurate, the results show that TIP4P/Ew and OPC are the models that capture more accurately the hydrogen-bond dynamics, though in the latter case, it might be via compensation of errors since OPC does not correctly describe the frequency fluctuations (see previous section).

Finally, it is also crucial to address the impact of the classical approximation in the conformational sampling. Nuclear quantum effects (NQEs), such as zero-point energy and tunneling, play a non-negligible role in liquid water dynamics.^117^ In particular, NQEs typically soften the hydrogen-bond network, allowing for greater proton delocalization than that predicted by classical models. In the context of our MD-IFM results, frequency fluctuation amplitudes are likely impacted, and an increase in it is expected due to the broader sampling of the potential energy surface, when quantum nuclear effects are included. Also, the τ_*c*_ values may be altered, as quantum fluctuations facilitate the reconfiguration of the hydrogen-bond network and lower the effective barriers for H-bond breaking and forming.^117^

## Conclusions

This study shows that the MD-IFM method is a robust tool for the benchmarking of molecular FFs. In particular, MD-IFM is successfully used to evaluate a series of common nonpolarizable water models, using MB-pol as a gold standard, and provides a unique insight into the accuracy of the FF for representing the structural and dynamics of liquid water. To this end, the evaluated FFs were: TIP3P, SPC/E, TIP4P/Ew, TIP5P, and OPC. All of these FFs performed reasonably well in predicting the average vibrational frequencies, suggesting a generally correct description of the average local structure experienced by a water molecule. The analysis of underlying distributions based on the number of water molecules in the first solvation shell of the probed water molecule confirmed that every force field consistently reproduces the expected vibrational solvatochromism due to changes in the hydrogen-bonding state. However, an analysis of the frequency distributions uncovered a systematic underestimation of the inherent heterogeneity of the water hydrogen-bond network. This error was particularly pronounced for the OPC model. In terms of the dynamics, all classical FFs exhibited faster dynamics of the vibrational frequency decorrelation in comparison to that of MB-pol showcasing the deficiency of the classical models for describing the hydrogen-bond rearrangements. While this study shows that there is not a clear victory among FFs, more complex 4-site and 5-site models tend to perform better at describing the chemical environment dynamics. Overall, we highlight the MD-IFM method as a valuable tool for assessing classical force fields through nonexpensive calculations of vibrational observables that account for the chemical environment structure and dynamics.

## Supplementary Material



## Notes

Details on Experimental Methods and Supporting figures and tables containing radial distribution functions for the water simulations, distributions of water molecules in the first solvation shell of the probe, frequency distributions including their fitting parameters, skewness, and contributions from subdistributions according to the number of water molecules in the first solvation shell, and FFCF and their corresponding fitting parameters (PDF)

## References

[1] Ren S., Liu X., Lin P., Gao Y., Erkens S. (2022). Molecular Dynamics
Simulation on Bulk Bitumen Systems and Its Potential Connections to
Macroscale Performance: Review and Discussion. Fuel.

[2] Li K., Clarkson C. M., Wang L., Liu Y., Lamm M., Pang Z., Zhou Y., Qian J., Tajvidi M., Gardner D. J., Tekinalp H., Hu L., Li T., Ragauskas A. J., Youngblood J. P., Ozcan S. (2021). Alignment of Cellulose
Nanofibers: Harnessing Nanoscale Properties to Macroscale Benefits. ACS Nano.

[3] Li G., Monroe C. W. (2020). Multiscale Lithium-Battery Modeling from Materials
to Cells. Annu. Rev. Chem. Biomol. Eng..

[4] Fecker T., Galaz-Davison P., Engelberger F., Narui Y., Sotomayor M., Parra L. P., Ramírez-Sarmiento C. A. (2018). Active Site Flexibility
as a Hallmark for Efficient PET Degradation by I. Sakaiensis PETase. Biophys. J..

[5] Lu S., Hu L., Lin H., Judge A., Rivera P., Palaniappan M., Sankaran B., Wang J., Prasad B. V. V., Palzkill T. (2022). An Active
Site Loop Toggles between Conformations to Control Antibiotic Hydrolysis
and Inhibition Potency for CTX-M β-Lactamase Drug-Resistance
Enzymes. Nat. Commun..

[6] Cui Y., Kuroda D. G. (2018). Evidence of Molecular Heterogeneities in Amide-Based
Deep Eutectic Solvents. J. Phys. Chem. A.

[7] Carvalho P. M., Felício M. R., Santos N. C., Gonçalves S., Domingues M. M. (2018). Application
of Light Scattering Techniques to Nanoparticle
Characterization and Development. Front. Chem..

[8] Chaney T. P., Levin A. J., Schneider S. A., Toney M. F. (2022). Scattering Techniques
for Mixed Donor–Acceptor Characterization in Organic Photovoltaics. Mater. Horiz..

[9] Markova N., Cairns S., Jankevics-Jones H., Kaszuba M., Caputo F., Parot J. (2022). Biophysical Characterization
of Viral and Lipid-Based Vectors for
Vaccines and Therapeutics with Light Scattering and Calorimetric Techniques. Vaccines (Basel)..

[10] Hedayat H., Sayers C. J., Ceraso A., van Wezel J., Clark S. R., Dallera C., Cerullo G., Da Como E., Carpene E. (2021). Investigation of the Non-Equilibrium
State of Strongly
Correlated Materials by Complementary Ultrafast Spectroscopy Techniques. New J. Phys..

[11] Nihonyanagi S., Yamaguchi S., Tahara T. (2017). Ultrafast Dynamics at Water Interfaces
Studied by Vibrational Sum Frequency Generation Spectroscopy. Chem. Rev..

[12] Hashimoto H., Sugisaki M., Yoshizawa M. (2015). Ultrafast
Time-Resolved Vibrational
Spectroscopies of Carotenoids in Photosynthesis. Biochim. Biophys. Acta, Bioenerg..

[13] Fecko C. J., Eaves J. D., Loparo J. J., Tokmakoff A., Geissler P. L. (2003). Ultrafast Hydrogen-Bond Dynamics in the Infrared Spectroscopy
of Water. Science (1979).

[14] Ramasesha K., De Marco L., Mandal A., Tokmakoff A. (2013). Water Vibrations
Have Strongly Mixed Intra- and Intermolecular Character. Nat. Chem..

[15] De
Marco L., Ramasesha K., Tokmakoff A. (2013). Experimental
Evidence of Fermi Resonances in Isotopically Dilute Water from Ultrafast
Broadband IR Spectroscopy. J. Phys. Chem. B.

[16] Fecko C. J., Loparo J. J., Roberts S. T., Tokmakoff A. (2005). Local Hydrogen
Bonding Dynamics and Collective Reorganization in Water: Ultrafast
Infrared Spectroscopy of HOD/D 2O. J. Chem.
Phys..

[17] Maréchal Y. (1994). IR Spectroscopy
of an Exceptional H-Bonded Liquid: Water. J.
Mol. Struct..

[18] Cowan M. L., Bruner B. D., Huse N., Dwyer J. R., Chugh B., Nibbering E. T. J., Elsaesser T., Miller R. J. D. (2005). Ultrafast Memory
Loss and Energy Redistribution in the Hydrogen Bond Network of Liquid
H2O. Nature.

[19] Woutersen S., Bakker H. J. (1999). Resonant Intermolecular Transfer
of Vibrational Energy
in Liquid Water. Nature.

[20] van
der Post S. T., Hsieh C.-S., Okuno M., Nagata Y., Bakker H. J., Bonn M., Hunger J. (2015). Strong Frequency Dependence
of Vibrational Relaxation in Bulk and Surface Water Reveals Sub-Picosecond
Structural Heterogeneity. Nat. Commun..

[21] De
Marco L., Fournier J. A., Thämer M., Carpenter W., Tokmakoff A. (2016). Anharmonic Exciton Dynamics and Energy
Dissipation in Liquid Water from Two-Dimensional Infrared Spectroscopy. J. Chem. Phys..

[22] Nienhuys H.-K., van Santen R. A., Bakker H. J. (2000). Orientational Relaxation of Liquid
Water Molecules as an Activated Process. J.
Chem. Phys..

[23] Asbury J.
B., Steinel T., Stromberg C., Corcelli S. A., Lawrence C. P., Skinner J. L., Fayer M. D. (2004). Water Dynamics: Vibrational Echo
Correlation Spectroscopy and Comparison to Molecular Dynamics Simulations. J. Phys. Chem. A.

[24] Loparo J. J., Roberts S. T., Nicodemus R. A., Tokmakoff A. (2007). Variation
of the Transition Dipole Moment across the OH Stretching Band of Water. Chem. Phys..

[25] Nicodemus R. A., Corcelli S. A., Skinner J. L., Tokmakoff A. (2011). Collective
Hydrogen Bond Reorganization in Water Studied with Temperature-Dependent
Ultrafast Infrared Spectroscopy. J. Phys. Chem.
B.

[26] Imoto S., Xantheas S. S., Saito S. (2013). Ultrafast
Dynamics of Liquid Water:
Frequency Fluctuations of the OH Stretch and the HOH Bend. J. Chem. Phys..

[27] De
Marco L., Carpenter W., Liu H., Biswas R., Bowman J. M., Tokmakoff A. (2016). Differences in the Vibrational Dynamics
of H _2_ O and D _2_ O: Observation of Symmetric
and Antisymmetric Stretching Vibrations in Heavy Water. J. Phys. Chem. Lett..

[28] Ojha D., Karhan K., Kühne T. D. (2018). On the
Hydrogen Bond Strength and
Vibrational Spectroscopy of Liquid Water. Sci.
Rep..

[29] Lascoux N., Gallot G., Hache F., Gale G. M., Bratos S., Leicknam J. (2000). Femtosecond Dynamics
of Hydrogen Bonds in Liquid Water:
A Real Time Study. J. Chin. Chem. Soc..

[30] Lawrence C. P., Skinner J. L. (2003). Vibrational Spectroscopy
of HOD in Liquid D2O. VI.
Intramolecular and Intermolecular Vibrational Energy Flow. J. Chem. Phys..

[31] Carpenter W. B., Fournier J. A., Biswas R., Voth G. A., Tokmakoff A. (2017). Delocalization
and Stretch-Bend Mixing of the HOH Bend in Liquid Water. J. Chem. Phys..

[32] Chuntonov L., Kumar R., Kuroda D. G. (2014). Non-Linear Infrared Spectroscopy
of the Water Bending Mode: Direct Experimental Evidence of Hydration
Shell Reorganization?. Phys. Chem. Chem. Phys..

[33] Rey R., Møller K. B., Hynes J. T. (2002). Hydrogen Bond Dynamics in Water and
Ultrafast Infrared Spectroscopy. J. Phys. Chem.
A.

[34] Lawrence C. P., Skinner J. L. (2002). Vibrational Spectroscopy
of HOD in Liquid D2O. I. Vibrational
Energy Relaxation. J. Chem. Phys..

[35] Lawrence C. P., Skinner J. L. (2002). Vibrational Spectroscopy
of HOD in Liquid D2O. II.
Infrared Line Shapes and Vibrational Stokes Shift. J. Chem. Phys..

[36] Lawrence C. P., Skinner J. L. (2003). Vibrational Spectroscopy of HOD in Liquid D2O. III.
Spectral Diffusion, and Hydrogen-Bonding and Rotational Dynamics. J. Chem. Phys..

[37] Møller K. B., Rey R., Hynes J. T. (2004). Hydrogen Bond Dynamics in Water and Ultrafast Infrared
Spectroscopy: A Theoretical Study. J. Phys.
Chem. A.

[38] Corcelli S.
A., Lawrence C. P., Skinner J. L. (2004). Combined Electronic Structure/Molecular
Dynamics Approach for Ultrafast Infrared Spectroscopy of Dilute HOD
in Liquid H 2O and D 2O. J. Chem. Phys..

[39] Corcelli S. A., Skinner J. L. (2005). Infrared and Raman
Line Shapes of Dilute HOD in Liquid
H _2_ O and D _2_ O from 10 to 90 °C. J. Phys. Chem. A.

[40] Auer B. M., Skinner J. L. (2008). IR and Raman Spectra of Liquid Water: Theory and Interpretation. J. Chem. Phys..

[41] Auer B. M., Skinner J. L. (2009). Water: Hydrogen
Bonding and Vibrational Spectroscopy,
in the Bulk Liquid and at the Liquid/Vapor Interface. Chem. Phys. Lett..

[42] Paesani F., Xantheas S. S., Voth G. A. (2009). Infrared Spectroscopy
and Hydrogen-Bond
Dynamics of Liquid Water from Centroid Molecular Dynamics with an
Ab Initio-Based Force Field. J. Phys. Chem.
B.

[43] Yang M., Skinner J. L. (2010). Signatures of Coherent
Vibrational Energy Transfer
in IR and Raman Line Shapes for Liquid Water. Phys. Chem. Chem. Phys..

[44] Shi L., Gruenbaum S. M., Skinner J. L. (2012). Interpretation of IR and Raman Line
Shapes for H _2_ O and D _2_ O Ice Ih. J. Phys. Chem. B.

[45] Wang Y., Bowman J. M. (2013). IR Spectra of the Water Hexamer: Theory, with Inclusion
of the Monomer Bend Overtone, and Experiment Are in Agreement. J. Phys. Chem. Lett..

[46] Ni Y., Skinner J. L. (2015). IR and SFG Vibrational Spectroscopy of the Water Bend
in the Bulk Liquid and at the Liquid-Vapor Interface, Respectively. J. Chem. Phys..

[47] Medders G.
R., Paesani F. (2015). Infrared and
Raman Spectroscopy of Liquid Water through
“First-Principles” Many-Body Molecular Dynamics. J. Chem. Theory Comput..

[48] Liu H., Wang Y., Bowman J. M. (2015). Quantum Calculations of the IR Spectrum
of Liquid Water Using *Ab Initio* and Model Potential
and Dipole Moment Surfaces and Comparison with Experiment. J. Chem. Phys..

[49] Medders G.
R., Paesani F. (2015). On the Interplay
of the Potential Energy and Dipole
Moment Surfaces in Controlling the Infrared Activity of Liquid Water. J. Chem. Phys..

[50] Straight S.
C., Paesani F. (2016). Exploring
Electrostatic Effects on the Hydrogen Bond
Network of Liquid Water through Many-Body Molecular Dynamics. J. Phys. Chem. B.

[51] Liu H., Wang Y., Bowman J. M. (2016). Transferable Ab Initio Dipole Moment
for Water: Three Applications to Bulk Water. J. Phys. Chem. B.

[52] Hunter K. M., Shakib F. A., Paesani F. (2018). Disentangling Coupling
Effects in
the Infrared Spectra of Liquid Water. J. Phys.
Chem. B.

[53] Nagata Y., Yoshimune S., Hsieh C.-S., Hunger J., Bonn M. (2015). Ultrafast
Vibrational Dynamics of Water Disentangled by Reverse Nonequilibrium *Ab Initio* Molecular Dynamics Simulations. Phys. Rev. X.

[54] Bakker H. J., Skinner J. L. (2010). Vibrational Spectroscopy
as a Probe of Structure and
Dynamics in Liquid Water. Chem. Rev..

[55] Perakis F., Marco L. D., Shalit A., Tang F., Kann Z. R., Kühne T. D., Torre R., Bonn M., Nagata Y. (2016). Vibrational
Spectroscopy and Dynamics of Water. Chem. Rev..

[56] Woutersen S., Emmerichs U., Nienhuys H.-K., Bakker H. (1998). Anomalous Temperature
Dependence of Vibrational Lifetimes in Water and Ice. Phys. Rev. Lett..

[57] Laenen R., Rauscher C., Laubereau A. (1998). Dynamics of
Local Substructures in
Water Observed by Ultrafast Infrared Hole Burning. Phys. Rev. Lett..

[58] Sepulveda-Montaño L. X., Galindo J. F., Kuroda D. G. (2024). A New Computational
Methodology for
the Characterization of Complex Molecular Environments Using IR Spectroscopy:
Bridging the Gap between Experiments and Computations. Chem. Sci..

[59] Leach, A. R. Molecular Modelling: Principles and Applications; Prentice Hall, 2001.

[60] Zhang H., Yin C., Jiang Y., van der Spoel D. (2018). Force Field Benchmark of Amino Acids:
I. Hydration and Diffusion in Different Water Models. J. Chem. Inf. Model..

[61] Zhang H., Jiang Y., Cui Z., Yin C. (2018). Force Field
Benchmark
of Amino Acids. 2. Partition Coefficients between Water and Organic
Solvents. J. Chem. Inf. Model..

[62] Sarthak K., Winogradoff D., Ge Y., Myong S., Aksimentiev A. (2023). Benchmarking
Molecular Dynamics Force Fields for All-Atom Simulations of Biological
Condensates. J. Chem. Theory Comput..

[63] Berg A., Peter C., Johnston K. (2017). Evaluation
and Optimization of Interface
Force Fields for Water on Gold Surfaces. J.
Chem. Theory Comput..

[64] Lee
Warren G., Patel S. (2007). Hydration Free Energies of Monovalent
Ions in Transferable Intermolecular Potential Four Point Fluctuating
Charge Water: An Assessment of Simulation Methodology and Force Field
Performance and Transferability. J. Chem. Phys..

[65] Lim V. T., Hahn D. F., Tresadern G., Bayly C. I., Mobley D. L. (2020). Benchmark
Assessment of Molecular Geometries and Energies from Small Molecule
Force Fields. F1000Res..

[66] Caleman C., van Maaren P. J., Hong M., Hub J. S., Costa L. T., van der Spoel D. (2012). Force Field Benchmark of Organic Liquids: Density,
Enthalpy of Vaporization, Heat Capacities, Surface Tension, Isothermal
Compressibility, Volumetric Expansion Coefficient, and Dielectric
Constant. J. Chem. Theory Comput..

[67] Boothroyd S., Behara P. K., Madin O. C., Hahn D. F., Jang H., Gapsys V., Wagner J. R., Horton J. T., Dotson D. L., Thompson M. W., Maat J., Gokey T., Wang L.-P., Cole D. J., Gilson M. K., Chodera J. D., Bayly C. I., Shirts M. R., Mobley D. L. (2023). Development
and Benchmarking of Open
Force Field 2.0.0: The Sage Small Molecule Force Field. J. Chem. Theory Comput..

[68] Błasiak B., Londergan C. H., Webb L. J., Cho M. (2017). Vibrational
Probes:
From Small Molecule Solvatochromism Theory and Experiments to Applications
in Complex Systems. Acc. Chem. Res..

[69] Hamm, P. ; Zanni, M. Concepts and Methods of 2D Infrared Spectroscopy; Cambridge University Press, 2011; . 10.1017/CBO9780511675935.

[70] Conte R., Botti G., Ceotto M. (2020). Sensitivity
of Semiclassical Vibrational
Spectroscopy to Potential Energy Surface Accuracy: A Test on Formaldehyde. Vib. Spectrosc..

[71] Baiz C. R., Błasiak B., Bredenbeck J., Cho M., Choi J. H., Corcelli S. A., Dijkstra A. G., Feng C. J., Garrett-Roe S., Ge N. H., Hanson-Heine M. W. D., Hirst J. D., Jansen T. L. C., Kwac K., Kubarych K. J., Londergan C. H., Maekawa H., Reppert M., Saito S., Roy S., Skinner J. L., Stock G., Straub J. E., Thielges M. C., Tominaga K., Tokmakoff A., Torii H., Wang L., Webb L. J., Zanni M. T. (2020). Vibrational Spectroscopic Map, Vibrational
Spectroscopy, and Intermolecular Interaction. Chem. Rev..

[72] Zhao R., Shirley J. C., Lee E., Grofe A., Li H., Baiz C. R., Gao J. (2022). Origin of
Thiocyanate Spectral Shifts
in Water and Organic Solvents. J. Chem. Phys..

[73] Wang L., Middleton C. T., Zanni M. T., Skinner J. L. (2011). Development and
Validation of Transferable Amide I Vibrational Frequency Maps for
Peptides. J. Phys. Chem. B.

[74] Gruenbaum S. M., Tainter C. J., Shi L., Ni Y., Skinner J. L. (2013). Robustness
of Frequency, Transition Dipole, and Coupling Maps for Water Vibrational
Spectroscopy. J. Chem. Theory Comput..

[75] Schmidt J. R., Roberts S. T., Loparo J. J., Tokmakoff A., Fayer M. D., Skinner J. L. (2007). Are Water Simulation
Models Consistent
with Steady-State and Ultrafast Vibrational Spectroscopy Experiments?. Chem. Phys..

[76] Hermansson K., Knuts S., Lindgren J. (1991). The OH Vibrational
Spectrum of Liquid
Water from Combined Ab Initio and Monte Carlo Calculations. J. Chem. Phys..

[77] Zhang X., Chen X., Kuroda D. G. (2021). Computing the Frequency Fluctuation
Dynamics of Highly Coupled Vibrational Transitions Using Neural Networks. J. Chem. Phys..

[78] Kwak K., Park S., Finkelstein I. J., Fayer M. D. (2007). Frequency-Frequency
Correlation Functions and Apodization in Two-Dimensional Infrared
Vibrational Echo Spectroscopy: A New Approach. J. Chem. Phys..

[79] Sepulveda-Montaño L. X., Galindo J. F., Kuroda D. G. (2023). Infrared Spectroscopy of Liquid Solutions
as a Benchmarking Tool of Semiempirical QM Methods: The Case of GFN2-XTB. J. Phys. Chem. B.

[80] Bannwarth C., Ehlert S., Grimme S. (2019). GFN2-XTB-An Accurate
and Broadly
Parametrized Self-Consistent Tight-Binding Quantum Chemical Method
with Multipole Electrostatics and Density-Dependent Dispersion Contributions. J. Chem. Theory Comput..

[81] Sepulveda-Montaño L. X., Galindo J. F., Kuroda D. G. (2025). Unraveling
the Heterogeneous but
Ordered Microstructure of the Nonionic Deep Eutectic Solvent Formed
by Lauric Acid and *N* -Methylacetamide. J. Phys. Chem. B.

[82] Jorgensen W. L., Chandrasekhar J., Madura J. D., Impey R. W., Klein M. L. (1983). Comparison
of Simple Potential Functions for Simulating Liquid Water. J. Chem. Phys..

[83] Berendsen H. J. C., Grigera J. R., Straatsma T. P. (1987). The Missing Term in Effective Pair
Potentialst. J. Phys. Chem..

[84] Allen, M. P. ; Tildesley, D. J. Computer Simulation of Liquids; Oxford University PressOxford, 2017; . 10.1093/oso/9780198803195.001.0001.

[85] Horn H. W., Swope W. C., Pitera J. W., Madura J. D., Dick T. J., Hura G. L., Head-Gordon T. (2004). Development
of an Improved Four-Site
Water Model for Biomolecular Simulations: TIP4P-Ew. J. Chem. Phys..

[86] Horn H. W., Swope W. C., Pitera J. W. (2005). Characterization of the TIP4P-Ew
Water Model: Vapor Pressure and Boiling Point. J. Chem. Phys..

[87] Izadi S., Anandakrishnan R., Onufriev A. V. (2014). Building Water Models: A Different
Approach. J. Phys. Chem. Lett..

[88] Mahoney M. W., Jorgensen W. L. (2000). A Five-Site
Model for Liquid Water and the Reproduction
of the Density Anomaly by Rigid, Nonpolarizable Potential Functions. J. Chem. Phys..

[89] Kříž K., van der Spoel D. (2024). Quantification of Anisotropy in Exchange and Dispersion
Interactions: A Simple Model for Physics-Based Force Fields. J. Phys. Chem. Lett..

[90] Shi Y., Xia Z., Zhang J., Best R., Wu C., Ponder J. W., Ren P. (2013). Polarizable Atomic Multipole-Based
AMOEBA Force Field for Proteins. J. Chem. Theory
Comput..

[91] Ponder J.
W., Wu C., Ren P., Pande V. S., Chodera J. D., Schnieders M. J., Haque I., Mobley D. L., Lambrecht D. S., DiStasio R. A., Head-Gordon M., Clark G. N. I., Johnson M. E., Head-Gordon T. (2010). Current Status of the AMOEBA Polarizable Force Field. J. Phys. Chem. B.

[92] Aytenfisu A. H., Yang M., MacKerell A. D. (2018). CHARMM Drude Polarizable Force Field
for Glycosidic Linkages Involving Pyranoses and Furanoses. J. Chem. Theory Comput..

[93] Nicholson B. F., Clancy P., Rick S. W. (2006). The Interface Response
Function and
Melting Point of the Prism Interface of Ice Ih Using a Fluctuating
Charge Model (TIP4P-FQ). J. Cryst. Growth.

[94] Caldwell J. W., Kollman P. A. (1995). Structure and Properties
of Neat Liquids Using Nonadditive
Molecular Dynamics: Water, Methanol, and N-Methylacetamide. J. Phys. Chem..

[95] Warshel A., Kato M., Pisliakov A. V. (2007). Polarizable Force Fields: History,
Test Cases, and Prospects. J. Chem. Theory Comput..

[96] Lopes P. E. M., Roux B., MacKerell A. D. (2009). Molecular
Modeling and Dynamics Studies
with Explicit Inclusion of Electronic Polarizability: Theory and Applications. Theor. Chem. Acc..

[97] Babin V., Leforestier C., Paesani F. (2013). Development of a “First
Principles”
Water Potential with Flexible Monomers: Dimer Potential Energy Surface,
VRT Spectrum, and Second Virial Coefficient. J. Chem. Theory Comput..

[98] Babin V., Medders G. R., Paesani F. (2014). Development of a “First Principles”
Water Potential with Flexible Monomers. II: Trimer Potential Energy
Surface, Third Virial Coefficient, and Small Clusters. J. Chem. Theory Comput..

[99] Medders G. R., Babin V., Paesani F. (2014). Development of a “First-Principles”
Water Potential with Flexible Monomers. III. Liquid Phase Properties. J. Chem. Theory Comput..

[100] Paesani F. (2016). Getting the
Right Answers for the Right Reasons: Toward
Predictive Molecular Simulations of Water with Many-Body Potential
Energy Functions. Acc. Chem. Res..

[101] Abascal J. L. F., Sanz E., García
Fernández R., Vega C. (2005). A Potential Model for the Study of
Ices and Amorphous Water: TIP4P/Ice. J. Chem.
Phys..

[102] Zhu X., Riera M., Bull-Vulpe E. F., Paesani F. M. B.-P. (2023). Sub-Chemical
Accuracy for Water Simulations from the Gas to the Liquid Phase. J. Chem. Theory Comput..

[103] Maxson T., Szilvási T. (2024). Transferable Water Potentials Using
Equivariant Neural Networks. J. Phys. Chem.
Lett..

[104] O’Neill N., Shi B. X., Baldwin W. J., Witt W. C., Csányi G., Gale J. D., Michaelides A., Schran C. (2025). Towards Routine Condensed
Phase Simulations with Delta-Learned
Coupled Cluster Accuracy: Application to Liquid Water. J. Chem. Theory Comput..

[105] Reddy S. K., Straight S. C., Bajaj P., Huy Pham C., Riera M., Moberg D. R., Morales M. A., Knight C., Götz A. W., Paesani F. (2016). On the Accuracy of the MB-Pol Many-Body
Potential for Water: Interaction Energies, Vibrational Frequencies,
and Classical Thermodynamic and Dynamical Properties from Clusters
to Liquid Water and Ice. J. Chem. Phys..

[106] Gartner T. E., Hunter K. M., Lambros E., Caruso A., Riera M., Medders G. R., Panagiotopoulos A. Z., Debenedetti P. G., Paesani F. (2022). Anomalies and Local Structure of
Liquid Water from Boiling to the Supercooled Regime as Predicted by
the Many-Body MB-Pol Model. J. Phys. Chem. Lett..

[107] Reddy S. K., Moberg D. R., Straight S. C., Paesani F. (2017). Temperature-Dependent
Vibrational Spectra and Structure of Liquid Water from Classical and
Quantum Simulations with the MB-Pol Potential Energy Function. J. Chem. Phys..

[108] Jing Z., Liu C., Cheng S. Y., Qi R., Walker B. D., Piquemal J.-P., Ren P. (2019). Polarizable Force Fields
for Biomolecular Simulations: Recent Advances and Applications. Annu. Rev. Biophys..

[109] Lin F.-Y., Huang J., Pandey P., Rupakheti C., Li J., Roux B., MacKerell A. D. (2020). Further
Optimization and Validation
of the Classical Drude Polarizable Protein Force Field. J. Chem. Theory Comput..

[110] Case D. A., Aktulga H. M., Belfon K., Cerutti D. S., Cisneros G. A., Cruzeiro V. W. D., Forouzesh N., Giese T. J., Götz A. W., Gohlke H., Izadi S., Kasavajhala K., Kaymak M. C., King E., Kurtzman T., Lee T.-S., Li P., Liu J., Luchko T., Luo R., Manathunga M., Machado M. R., Nguyen H. M., O’Hearn K. A., Onufriev A. V., Pan F., Pantano S., Qi R., Rahnamoun A., Risheh A., Schott-Verdugo S., Shajan A., Swails J., Wang J., Wei H., Wu X., Wu Y., Zhang S., Zhao S., Zhu Q., Cheatham T. E., Roe D. R., Roitberg A., Simmerling C., York D. M., Nagan M. C., Merz K. M. (2023). AmberTools. J. Chem. Inf. Model..

[111] Martínez L., Andrade R., Birgin E. G., Martínez J. M. (2009). PACKMOL:
A Package for Building Initial Configurations for Molecular Dynamics
Simulations. J. Comput. Chem..

[112] Kropman M. F., Nienhuys H. K., Woutersen S., Bakker H. J. (2001). Vibrational Relaxation and Hydrogen-Bond Dynamics of
HDO:H2O. J. Phys. Chem. A.

[113] Auer B., Kumar R., Schmidt J. R., Skinner J. L. (2007). Hydrogen
Bonding and Raman, IR, and 2D-IR Spectroscopy of Dilute HOD in Liquid
D 2 O. Proc Natl Acad Sci U S A.

[114] Skinner J. L. (2008). Vibrational Line Shapes and Spectral Diffusion in Fluids. Mol. Phys..

[115] Šanda F., Perlík V., Lincoln C. N., Hauer J. (2015). Center Line
Slope Analysis in Two-Dimensional Electronic Spectroscopy. J. Phys. Chem. A.

[116] Lawrence C. P., Skinner J. L. (2003). Ultrafast Infrared Spectroscopy Probes
Hydrogen-Bonding Dynamics in Liquid Water. Chem.
Phys. Lett..

[117] Ceriotti M., Fang W., Kusalik P. G., McKenzie R. H., Michaelides A., Morales M. A., Markland T. E. (2016). Nuclear
Quantum
Effects in Water and Aqueous Systems: Experiment, Theory, and Current
Challenges. Chem. Rev..

