# Supplementary material for: Molecular Dynamics-Instantaneous Frequencies of Molecules Method to Address the Performance of Classical and Rigid Water Force Fields
Source: J Phys Chem B. 2026 Apr 29;130(19):5026–36. doi: 10.1021/acs.jpcb.5c08265 (PMC13181783; doi:10.1021/acs.jpcb.5c08265)
Supplement: Supplementary file 1 [file jp5c08265_si_001.pdf]

Supporting Information:

MD-IFM method to Address the Performance of Classical and Rigid Water Force Fields

Nicolas Molina Trujillo,[a] *Laura X. Sepulveda-Montaño,*[b] *Daniel G. Kuroda,*[b] *Johan F. Galindo**[a]

[a] Department of Chemistry, Universidad Nacional de Colombia sede Bogotá, 111321 Bogotá, Colombia.

[b] Department of Chemistry, Louisiana State University, Baton Rouge, Louisiana 70803, United States.

*Address correspondence to: jfgalindoc@unal.edu.co

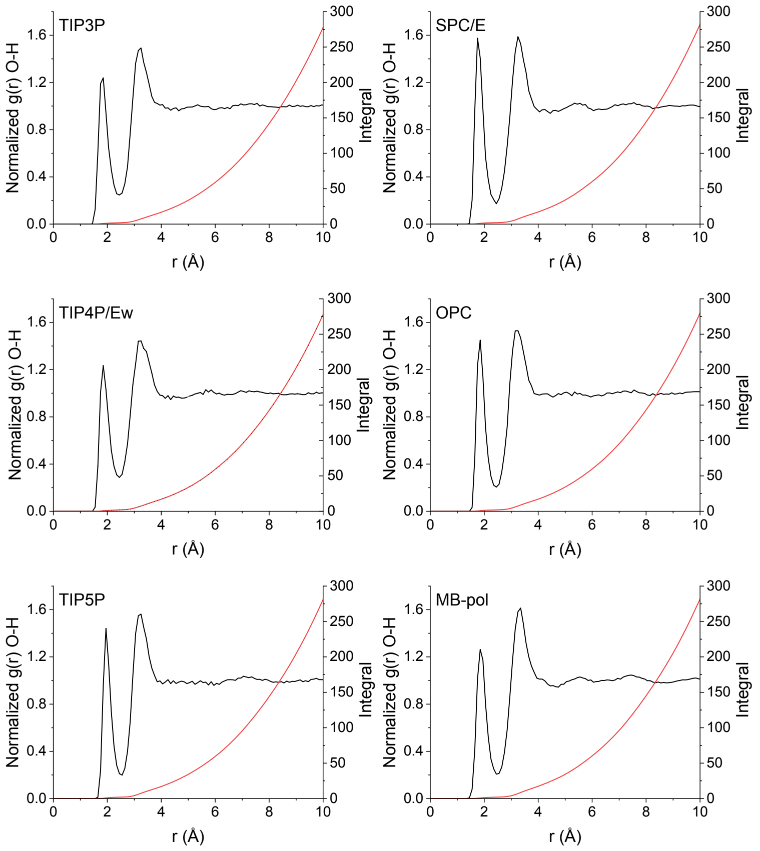

Figure S1. Radial distribution functions obtained from the simulations for every water force field.

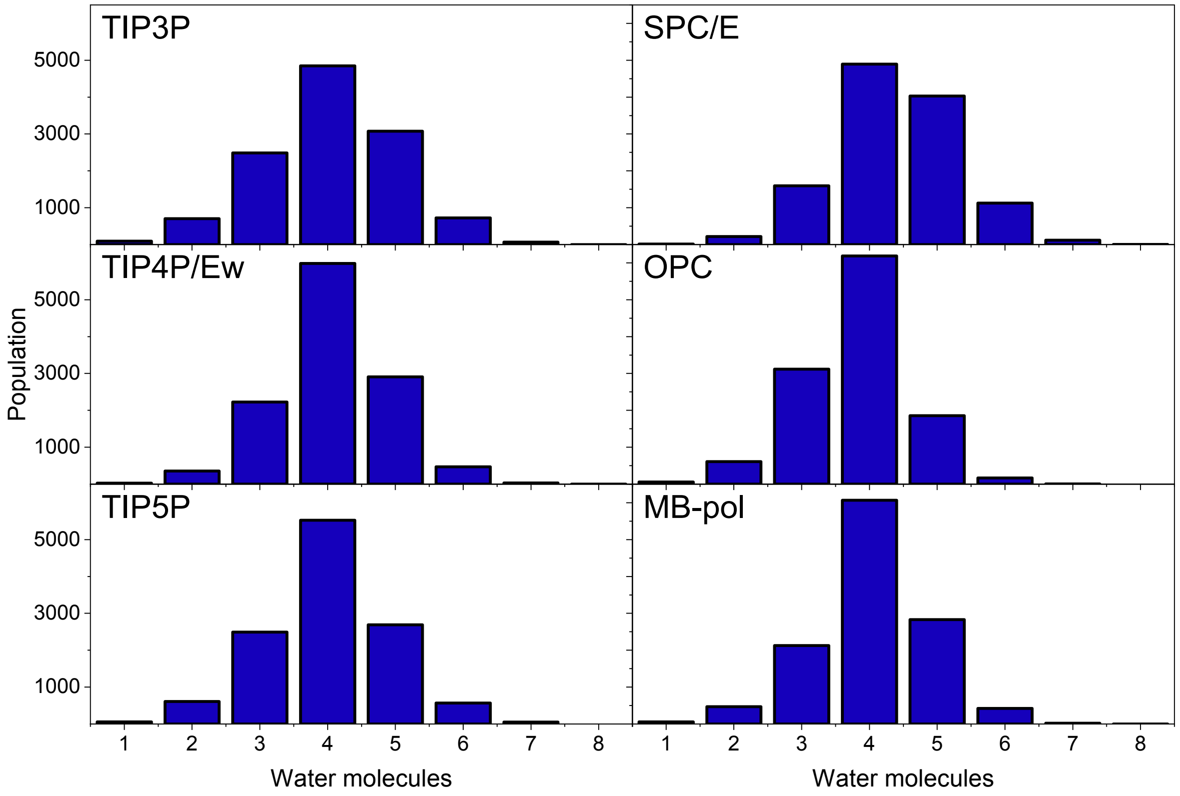

Figure S2. Distributions of water molecules in the first solvation shell of the probe throughout the simulations with every force field.

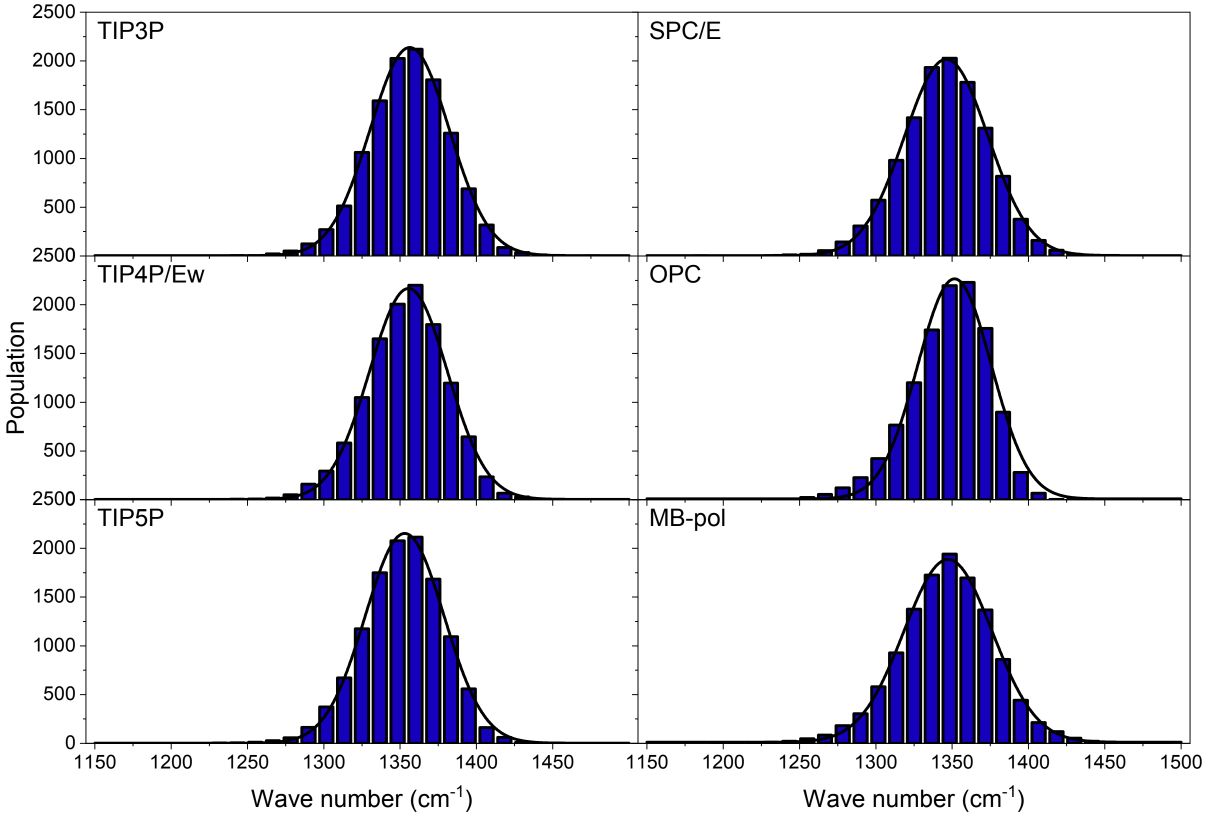

Figure S3. Frequency distributions of the HOD bending mode. Black line represents the Gaussian function fit (regression data is presented in table S1).

Table S1. Fitting parameters using a gaussian function for the HOD bending mode frequency distribution.

Force field	σ (cm^{-1})	Percentage errors respect MB-pol	R^2
TIP3P	26.0 ± 0.3	-10.22	0.999
SPC/E	27.4 ± 0.5	-5.13	0.999
TIP4P-Ew	25.5 ± 0.6	-11.63	0.999
OPC	24.2 ± 1.4	-16.41	0.998
TIP5P	25.8 ± 0.6	-10.65	0.998
MB-pol	28.9 ± 0.7	-	0.998

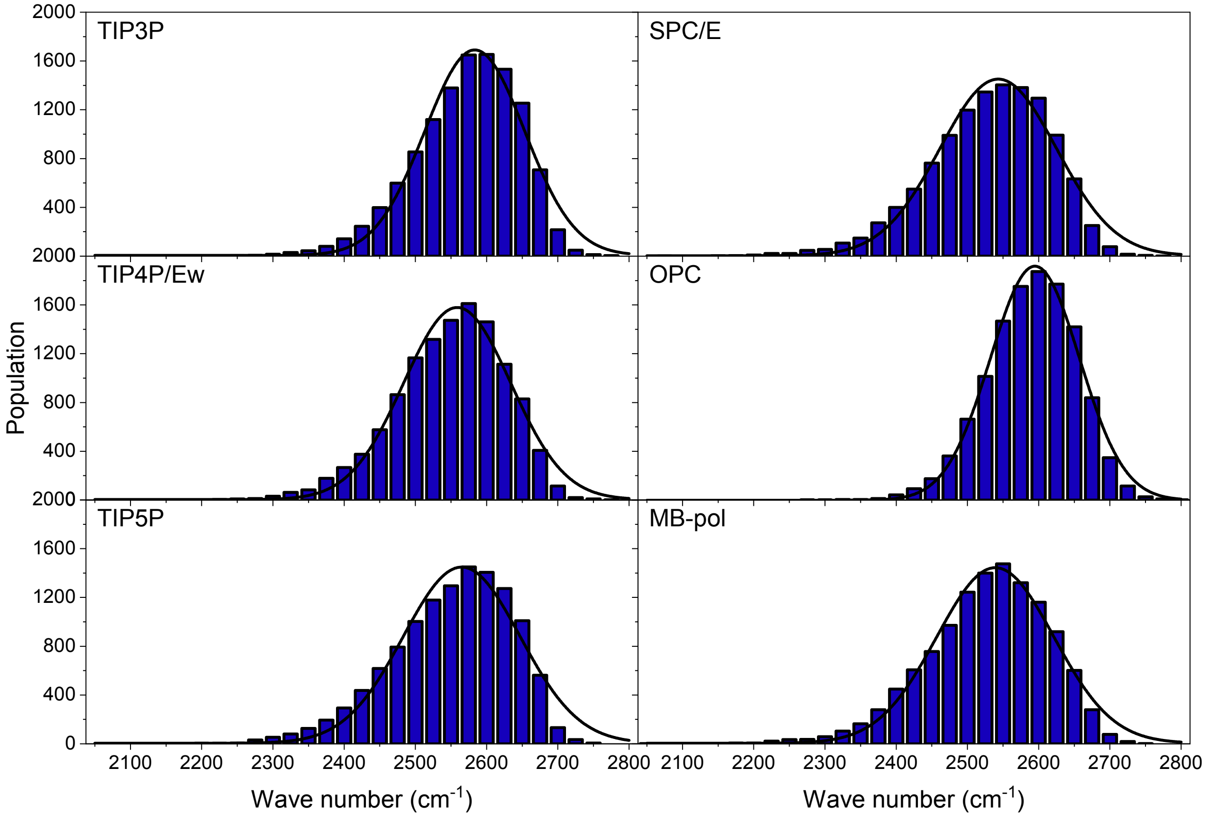

Figure S4. Frequency distributions of the OD stretching mode. Black line represents the Gaussian function fit (regression data is presented in table S2).

Table S2. Fitting parameters using a gaussian function for the OD stretching mode frequency distribution.

Force field	σ (cm^{-1})	Percentage errors respect MB-pol	R^2
TIP3P	70 ± 5	-14.80	0.980
SPC/E	82 ± 6	-0.27	0.982
TIP4P-Ew	75 ± 5	-8.43	0.987
OPC	63 ± 2	-23.84	0.994
TIP5P	82 ± 8	-0.20	0.968
MB-pol	82 ± 5	-	0.987

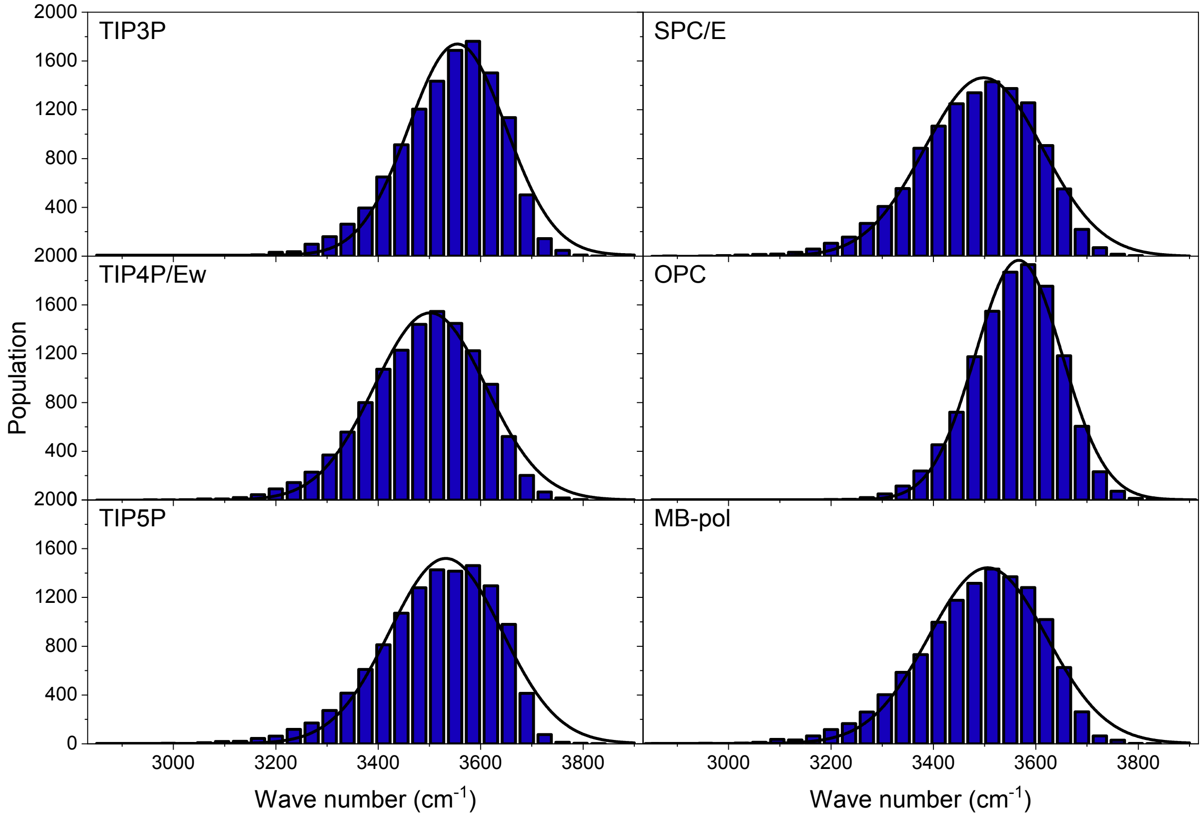

Figure S5. Frequency distributions for the OH stretching mode. Black line represents the Gaussian function fit (regression data is presented in table S3).

Table S3. Fitting parameters using a gaussian function for the OH stretching mode frequency distribution.

Force field	σ (cm^{-1})	Percentage errors respect MB-pol	R^2
TIP3P	95 ± 7	-17.68	0.979
SPC/E	115 ± 7	-0.34	0.985
TIP4P-Ew	109 ± 6	-5.45	0.988
OPC	85 ± 4	-26.46	0.993
TIP5P	110 ± 10	-4.86	0.971
MB-pol	115 ± 8	-	0.980

Table S4. Central frequencies of the HOD bending mode for the complete frequencies' distribution and the corresponding contribution from frames where the probe had 3, 4, 5 and 6 water molecules in its first solvation shell.

Force field	Central frequency (cm^{-1})				
	3 water molecules	4 water molecules	5 water molecules	6 water molecules	All frequencies
TIP3P	1351.3 ± 1.0	1351.4 ± 0.7	1349.4 ± 1.0	-	1350.2 ± 0.5
SPC/E	1342.0 ± 1.4	1340.1 ± 0.8	1338.9 ± 0.9	1339.4 ± 1.7	1339.8 ± 0.5
TIP4P-Ew	1351.3 ± 1.1	1348.8 ± 0.7	1346.8 ± 0.9	-	1348.5 ± 0.5
OPC	1342.9 ± 0.9	1340.6 ± 0.6	1340.4 ± 1.2	-	1341.2 ± 0.5
TIP5P	1347.5 ± 1.0	1346.4 ± 0.7	1345.0 ± 1.0	-	1346.1 ± 0.5
MB-pol	1343.0 ± 1.3	1341.8 ± 0.8	1338.1 ± 1.2	-	1340.9 ± 0.6

Table S5. Asymmetry coefficients of the HOD bending mode for the complete frequencies' distribution and the corresponding sub-distributions of the frames where the probe had 3, 4, 5 and 6 water molecules in its first solvation shell.

Force field	Asymmetry coefficient				
	3 water molecules	4 water molecules	5 water molecules	6 water molecules	All frequencies
TIP3P	-0.06	-0.16	-0.16	-	-0.15
SPC/E	-0.09	-0.14	-0.24	-0.32	-0.18
TIP4P-Ew	-0.23	-0.29	-0.27	-	-0.26
OPC	-0.58	-0.57	-0.58	-	-0.57
TIP5P	-0.12	-0.33	0.32	-	-0.27
MB-pol	-0.02	-0.16	-0.13	-	-0.14

Table S6. Central frequencies of the OD stretching mode for the complete frequencies' distribution and the corresponding contribution from frames where the probe had 3, 4, 5 and 6 water molecules in its first solvation shell.

Force field	Central frequency (cm^{-1})				
	3 water molecules	4 water molecules	5 water molecules	6 water molecules	All frequencies
TIP3P	2538 ± 3	2516 ± 2	2515 ± 3	-	2520.1 ± 1.5
SPC/E	2490 ± 5	2473 ± 2	2470 ± 3	2467 ± 5	2473.2 ± 1.6
TIP4P-Ew	2514 ± 4	2499 ± 2	2488 ± 3	-	2498.0 ± 1.5
OPC	2559 ± 2	2548 ± 2	2547 ± 3	-	1551.2 ± 1.2

TIP5P	2497 ± 4	2481 ± 2	2490 ± 3	-	2488.1 ± 1.6
MB-pol	2485 ± 4	2468 ± 2	2464 ± 3	-	2470.2 ± 1.6

Table S7. Asymmetry coefficients of the OD bending mode for the complete frequencies' distribution and the corresponding sub-distributions of the frames where the probe had 3, 4, 5 and 6 water molecules in its first solvation shell.

Force field	Asymmetry coefficient				
	3 water molecules	4 water molecules	5 water molecules	6 water molecules	All frequencies
TIP3P	-0.78	-0.59	-0.54	-	-0.63
SPC/E	-1.01	-0.58	-0.61	-0.61	-0.61
TIP4P-Ew	-0.87	-0.50	-0.64	-	-0.60
OPC	-0.44	-0.28	-0.33	-	-0.34
TIP5P	-1.04	-0.55	-0.54	-	-0.65
MB-pol	-0.87	-0.60	-0.53	-	-0.59

Table S8. Central frequencies of the OH stretching mode for the complete frequencies' distribution and the corresponding contribution from frames where the probe had 3, 4, 5 and 6 water molecules in its first solvation shell.

Force field	Central frequency (cm^{-1})				
	3 water molecules	4 water molecules	5 water molecules	6 water molecules	All frequencies
TIP3P	3476 ± 4	3469 ± 3	3461 ± 4	-	3468 ± 2
SPC/E	3424 ± 6	3405 ± 3	3404 ± 4	3393 ± 7	3406 ± 2
TIP4P-Ew	3442 ± 5	3416 ± 3	3406 ± 4	-	3418 ± 2
OPC	3521 ± 3	3503 ± 2	3494 ± 4	-	3507 ± 2
TIP5P	3437 ± 5	3422 ± 3	3423 ± 4	-	3426 ± 2
MB-pol	3436 ± 5	3397 ± 3	3393 ± 5	-	3403 ± 2

Table S9. Asymmetry coefficients of the OH stretching mode for the complete frequencies' distribution and the corresponding sub-distributions of the frames where the probe had 3, 4, 5 and 6 water molecules in its first solvation shell.

Force field	Asymmetry coefficient				
	3 water molecules	4 water molecules	5 water molecules	6 water molecules	All frequencies
TIP3P	-0.80	-0.57	-0.54		-0.62
SPC/E	-0.69	-0.51	-0.54	-0.55	-0.54
TIP4P-Ew	-0.56	-0.44	-0.56		-0.49
OPC	-0.41	-0.32	-0.29		-0.36
TIP5P	-1.01	-0.64	-0.58		-0.68
MB-pol	-0.72	-0.56	-0.68		-0.60

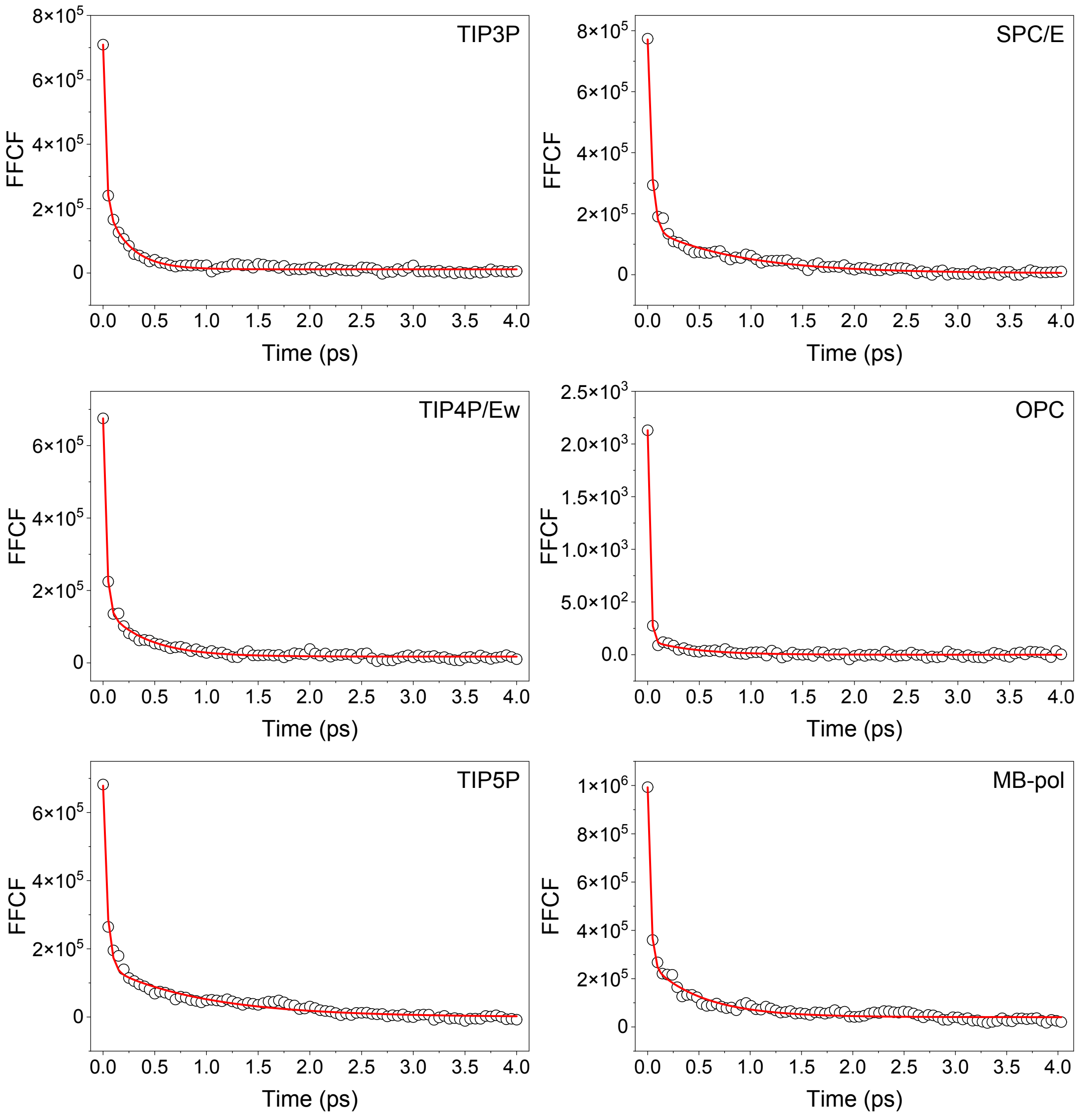

Figure S6. Frequency-frequency correlation function (FFCF) of the HOD bending mode with a bi-exponential decay function fit (red lines) for all the water force fields.

Table S10. Bi-exponential decay fitting parameters for the frequency-frequency correlation function (FFCF) of the HOD bending mode.

Force field	τ_1 (ps)	τ_c (ps)	R^2
TIP3P	0.030 ± 0.002	0.23 ± 0.02	0.993
SPC/E	0.034 ± 0.003	0.86 ± 0.07	0.984
TIP4P-Ew	0.031 ± 0.003	0.40 ± 0.03	0.986
OPC	0.029 ± 0.001	0.44 ± 0.07	0.995
TIP5P	0.031 ± 0.002	0.95 ± 0.05	0.990
MB-pol	0.027 ± 0.002	0.51 ± 0.04	0.988

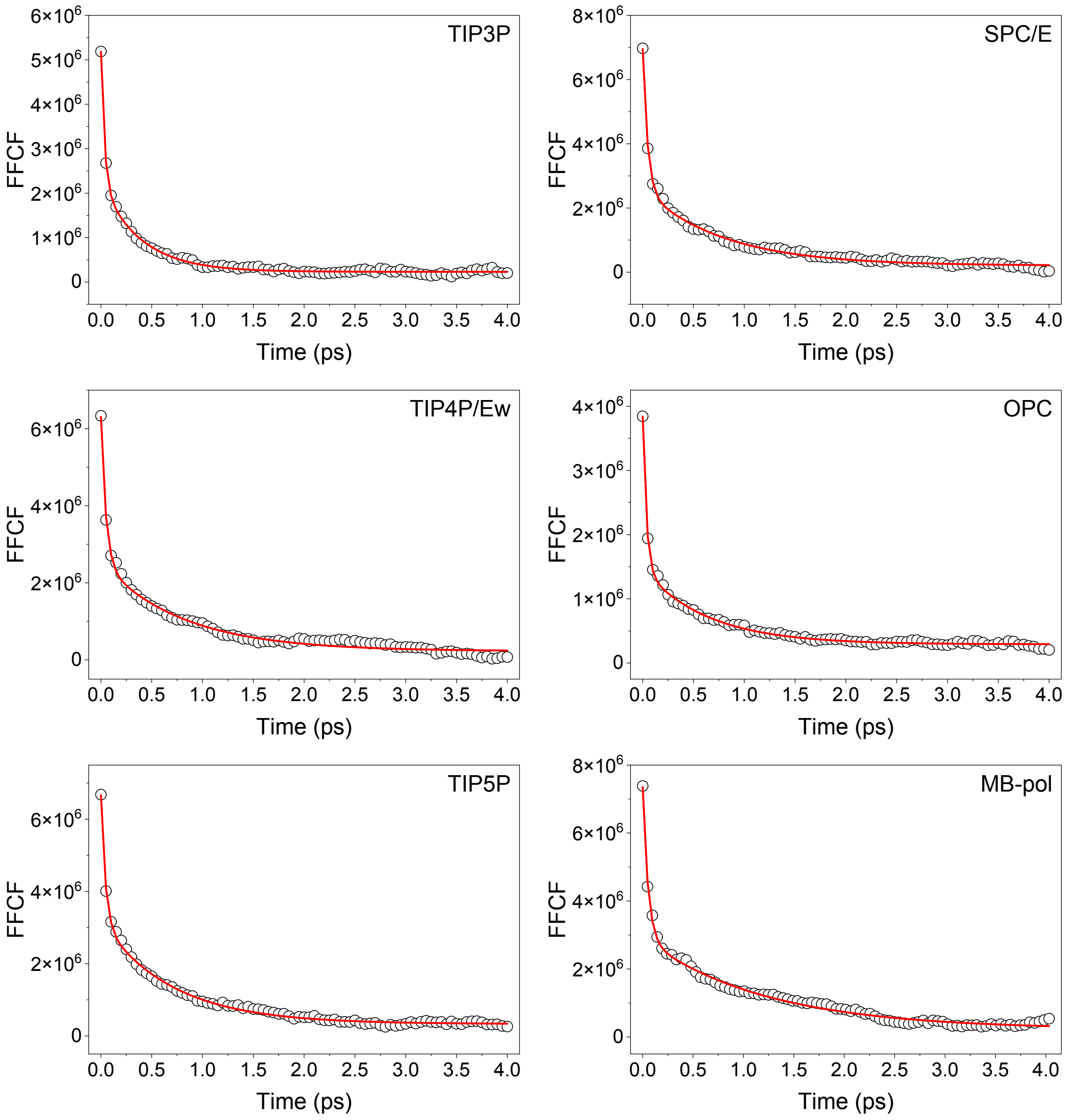

Figure S7. Frequency-frequency correlation function (FFCF) of the OD stretching mode with a bi-exponential decay function fit (red lines) for all the water force fields.

Table S11. Bi-exponential decay fitting parameters for the frequency-frequency correlation function (FFCF) of the OD stretching mode.

Force field	τ_1 (ps)	τ_c (ps)	R^2
TIP3P	0.037 ± 0.004	0.39 ± 0.01	0.989
SPC/E	0.050 ± 0.003	0.80 ± 0.04	0.992
TIP4P-Ew	0.037 ± 0.003	0.80 ± 0.05	0.994
OPC	0.040 ± 0.002	0.64 ± 0.02	0.995
TIP5P	0.054 ± 0.004	0.69 ± 0.02	0.991
MB-pol	0.055 ± 0.003	1.21 ± 0.06	0.994

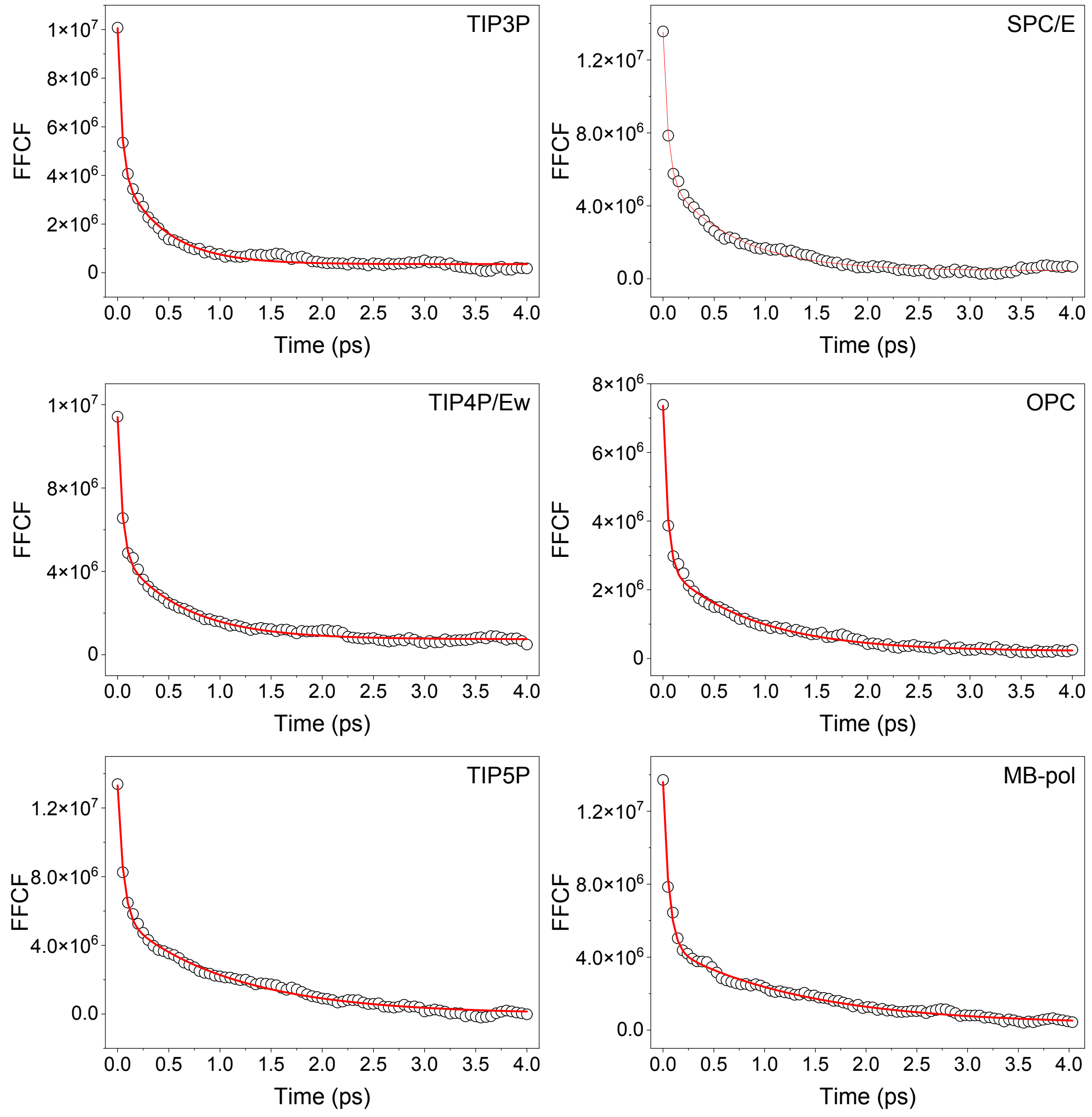

Figure S8. Frequency-frequency correlation function (FFCF) of the OH stretching mode with a bi-exponential decay function fit (red lines) for all the water force fields.

Table S12. Bi-exponential decay fitting parameters for the frequency-frequency correlation function (FFCF) of the OH stretching mode.

Force field	τ_1 (ps)	τ_c (ps)	R^2
TIP3P	0.035 ± 0.002	0.43 ± 0.03	0.996
SPC/E	0.049 ± 0.003	0.65 ± 0.03	0.991
TIP4P-Ew	0.050 ± 0.004	0.83 ± 0.05	0.998
OPC	0.040 ± 0.002	0.85 ± 0.04	0.993
TIP5P	0.045 ± 0.002	1.09 ± 0.03	0.996
MB-pol	0.055 ± 0.003	1.34 ± 0.08	0.992
